# ALYREF, a novel factor involved in breast carcinogenesis, acts through transcriptional and post-transcriptional mechanisms selectively regulating the short NEAT1 isoform

**DOI:** 10.1007/s00018-022-04402-2

**Published:** 2022-07-01

**Authors:** Christiane Klec, Erik Knutsen, Daniela Schwarzenbacher, Katharina Jonas, Barbara Pasculli, Ellen Heitzer, Beate Rinner, Katarina Krajina, Felix Prinz, Benjamin Gottschalk, Peter Ulz, Alexander Deutsch, Andreas Prokesch, Stephan W. Jahn, S. Mohammad Lellahi, Maria Perander, Raffaela Barbano, Wolfgang F. Graier, Paola Parrella, George Adrian Calin, Martin Pichler

**Affiliations:** 1 Division of Oncology, Department of Internal Medicine, Medical University of Graz, Augenbruggerplatz 15, 8010 Graz, Austria; 2 Research Unit for Non-Coding RNAs and Genome Editing, Medical University of Graz (MUG), Graz, Austria; 3 Department of Experimental Therapeutics, The University of Texas MD Anderson Cancer Center, Houston, TX 77030 USA; 4 Department of Medical Biology, Faculty of Health Sciences, UiT–the Arctic University of Norway, Tromsö, Norway; 5 Fondazione IRCCS Casa Sollievo della Sofferenza Laboratorio di Oncologia, San Giovanni Rotondo, FG Italy; 6 Institute of Human Genetics, Medical University of Graz (MUG), Graz, Austria; 7 Biomedical Research, Medical University of Graz (MUG), Graz, Austria; 8 Molecular Biology and Biochemistry, Gottfried Schatz Research Center for Cellular Signaling, Metabolism and Aging, Medical University of Graz (MUG), Graz, Austria; 9 Division of Hematology, Department of Internal Medicine, Medical University of Graz (MUG), Graz, Austria; 10 Division of Cell Biology, Histology and Embryology, Gottfried Schatz Research Center for Cell Signaling, Metabolism and Aging, Medical University of Graz, Graz, Austria; 11 Institute of Pathology, Diagnostic and Research Center for Molecular BioMedicine, Medical University of Graz, Graz, Austria; 12 Department of Translational Pathology, The University of Texas MD Anderson Cancer Center, Houston, TX USA

**Keywords:** Breast cancer, ALYREF, Transcriptional regulation, lncRNA, *NEAT1*

## Abstract

The RNA-binding protein ALYREF (THOC4) is involved in transcriptional regulation and nuclear mRNA export, though its role and molecular mode of action in breast carcinogenesis are completely unknown. Here, we identified high ALYREF expression as a factor for poor survival in breast cancer patients. ALYREF significantly influenced cellular growth, apoptosis and mitochondrial energy metabolism in breast cancer cells as well as breast tumorigenesis in orthotopic mouse models. Transcriptional profiling, phenocopy and rescue experiments identified the short isoform of the lncRNA *NEAT1* as a molecular trigger for ALYREF effects in breast cancer. Mechanistically, we found that ALYREF binds to the *NEAT1* promoter region to enhance the global *NEAT1* transcriptional activity. Importantly, by stabilizing CPSF6, a protein that selectively activates the post-transcriptional generation of the short isoform of *NEAT1*, as well as by direct binding and stabilization of the short isoform of *NEAT1,* ALYREF selectively fine-tunes the expression of the short *NEAT1* isoform. Overall, our study describes ALYREF as a novel factor contributing to breast carcinogenesis and identifies novel molecular mechanisms of regulation the two isoforms of *NEAT1*.

**Supplementary Information:**

The online version contains supplementary material available at 10.1007/s00018-022-04402-2.

## Introduction

Breast cancer is the leading cause of cancer-related deaths in women aged 20–60 years. Approximately 276,480 cases of female breast cancer were expected to be diagnosed in the United States in 2020, and breast cancer alone is estimated to account for 30% of all new cancer diagnoses in women [1]. In general, breast cancer is a very heterogeneous disease in terms of underlying biology, treatment response and prognosis, and it is commonly classified into several subtypes based on gene expression profiles or simplified into three major subtypes based on the presence or absence of immunohistochemical markers [2, 3]. Triple-negative breast cancer (TNBC, i.e., negative for estrogen receptor, progesterone receptor and HER2 protein) is the most aggressive breast cancer subtype, with poor prognosis due to limited therapeutic options [4, 5]. Though the majority of patients receive cytotoxic chemotherapy, progress in understanding the underlying biology of TNBC has led to the introduction of poly(ADP-ribose) polymerase (PARP) inhibitors, Trop-2 directed antibody drug conjugates and immune checkpoint inhibitors in certain patient cohorts [6–8]. Thus, a more profound understanding of the molecular mechanisms involved in TNBC formation is of paramount importance to improve the clinical outcome of those patients and ensure the development of novel and more effective cancer treatments.

The RNA-binding protein ALYREF (also called THOC4) was originally discovered as a partner of the TRanscription EXport (TREX) protein complex that binds to spliced mRNAs and enables transfer to the cytoplasm [9–11]. As part of the nuclear export TREX complex, ALYREF acts as an mRNA export adaptor by mediating the interaction between the mRNA and the mRNA export receptor nuclear RNA export factor 1 (NXF1) [12]. ALYREF has been demonstrated to mainly bind to the 5’ and 3’ ends of mRNA in vivo to facilitate this nuclear export [13]. 5-methylcytosine (m^5^C) additions to RNAs display a crucial modification of RNAs important for nuclear export [14]. m^5^C formation in mRNAs is mainly catalyzed by the RNA methyltransferase NOP2/Sun RNA Methyltransferase 2 (NSUN2). These m^5^C additions are recognized by ALYREF, initiating nuclear mRNA export [15]. The dysregulation of m^5^C changes contribute to the development of cancerous and non-cancerous diseases. As a m^5^C-reader, ALYREF has been demonstrated to contribute to pathogenesis of bladder cancer [16] and hepatocellular carcinoma development [14]. In addition to nuclear export factor capacities, ALYREF has been described as a transcriptional co-activator for basic region-leucin zipper transcription factors, including the regulation of erythropoiesis and leukemogenesis [17, 18]. Furthermore, ALYREF crucially contributes to genomic stability by suppressing R-loop (RNA–DNA hybrids) formation [19]. ALYREF expression is dysregulated in primary tumors [20] and has been linked to cellular proliferation and mRNA export through selective regulation of the S and G2/M phases by nuclear AKT phosphorylation [21] as well as the regulation of stability of MYC family members in neuro- and glioblastoma [22, 23]. As there are no systematic studies of ALYREF and its role in human breast cancer, we aimed for the first time to explore and comprehensively characterize the putative role of this RNA-binding protein and transcriptional activator in human breast carcinogenesis.

## Materials and methods

### Patient cohort/clinical data

To explore possible genomic alterations of the ALYREF gene, we made use of publicly available data (www.cbioportal.com version 3.0.2) [24, 25]. Four patient cohorts comprising 3989 patients with available data for chromosomal alterations were included in this analysis (including the 2509 samples of the METABRIC cohort, 1084 samples of the TCGA-PanCancer Atlas, 216 samples of the INSERM dataset and 237 samples of the www.mbcproject.org). A cohort of 128 female breast cancer patients with ALYREF mRNA expression data was provided by the Laboratory of Oncology, IRCCS Casa Sollievo della Sofferenza, Viale Padre Pio, 71013 San Giovanni Rotondo, (FG), Italy. RNA preparation and quantitative PCR were performed in this laboratory as previously described [26, 27]. The relative expression levels of ALYREF were determined by qRT-PCR. To perform confirmation in a second cohort, we made use of the publicly available dataset using the online tool (http://kmplot.com) to analyze microarray-derived data of 1764 breast cancer patients from different cohorts [28]. mRNA expression levels of patient samples were derived from the publicly available database https://tnmplot.com/analysis/, breast cancer subtype-specific expression data as well as correlation data were derived from http://bcgenex.ico.unicancer.fr/BC-GEM/GEM-Accueil.php?js=1 and cell line correlation data from https://depmap.org/portal/.

### In vitro and in vivo functional assays

Detailed protocols for phenotypic experiments (cellular growth assays, apoptosis assays, and mammosphere formation), functional experiments (luciferase reporter assay, RNA immunoprecipitation, chromatin immunoprecipitation, and mRNA stability assays, cycloheximide chase assay), imaging protocols and in vivo tumor growth in a xenograft mouse model can be found in the Supplementary Materials and methods section.

### Statistics and reproducibility

All statistical analyses were performed using SPSS version 20 software (SPSS Inc., Chicago, IL, USA) or GraphPad Prism 5.0 (GraphPad Software, La Jolla, CA, USA).

Data shown represent mean ± SEM/SD. ‘*n*’ values refer to the number of individual experiments performed. If applicable analysis of variance (ANOVA) was used for data evaluation and statistical significance of differences between means was estimated by Bonferroni post hoc test or two-tailed Student’s *t* test assuming unequal variances was used, where applicable using GraphPad Prism 5.0 (GraphPad Software, La Jolla, CA, USA).

## Results

### ALYREF gene is amplified across human cancers, and high mRNA and protein expression levels are associated with poor survival in breast cancer patients

**Fig. 1:**
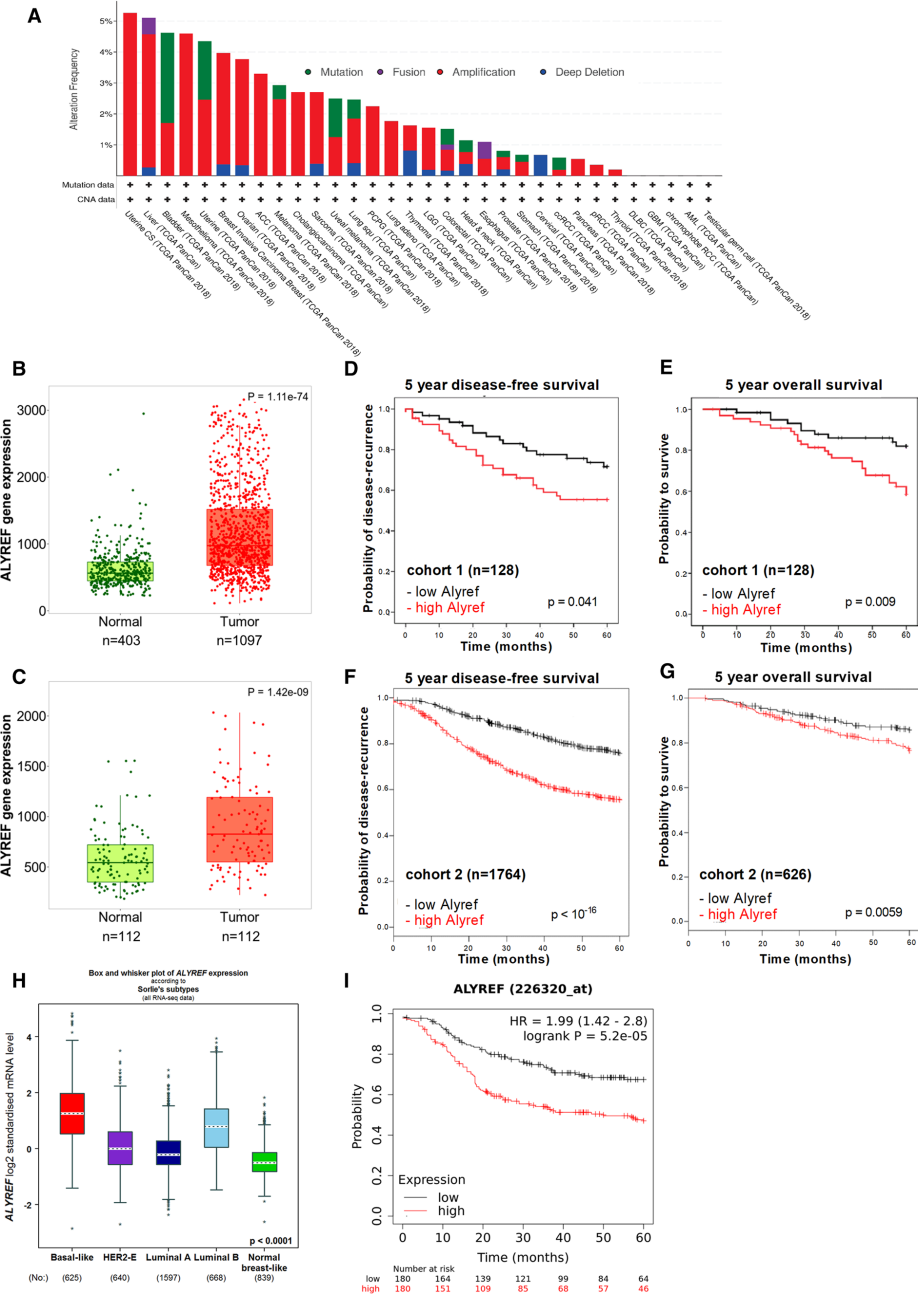
ALYREF gene amplifications across human cancers, expression levels are increased in breast cancer tissue and negatively impacts patient survival. **A** Genomic alterations of ALYREF in 32 cancer types of more than 10,000 patients indicates gene amplifications in a variety of cancer types including 4% of breast cancer patients. **B**, **C** Data showing ALYREF expression in unmatched tissue samples in A and matched tissue samples in B indicating a significant up-regulation in breast cancer tissue. RNA-seq data derived from the Cancer Genome Atlas. High ALYREF expression levels are associated with poor disease-free (**D**) (*n* = 128; *p* = 0.041, log-rank test) and overall survival (**E**) (*n* = 128; *p* = 0.009, log-rank test) in cohort 1. Confirmation of high ALYREF levels with poor disease-free (**F**) (*n* = 1764; *p* < 0.001) and overall survival (**G**) (*n* = 626; *p* = 0.0059) in an independent dataset of cohort 2. **H** Expression levels in different subtypes of breast cancer show highest ALYREF expression levels in basal-like breast cancer subtype. Statistical analysis can be found in Table S3. **I** High ALYREF expression is associated with poor recurrence-free survival of patients with triple-negative breast cancer subtype (*n* = 360; *p* < 0.0001)

To clarify the human relevance of ALYREF expression in breast cancer, we explored chromosomal alterations of the ALYREF gene region in more than 10,000 patients throughout 32 cancer types in the TCGA-PanCancer Atlas dataset. The most frequently found genomic alteration for this gene region was a focal amplification across different cancer types including 4% of breast cancer patients (Fig. 1A). Focusing more on breast cancer, we analyzed data from 3989 patients from four breast cancer cohorts and detected an average of 5% ALYREF gene amplification frequency (range 3.6–6.94% amplified cases, Fig. S1A). Fitting to the gain of genetic information, we observed increased ALYREF mRNA expression in breast tumor tissue when compared to normal breast tissue (Fig. 1B) as well as in matching normal and cancerous breast tissue samples (Fig. 1C) from TCGA datasets (https://tnmplot.com/analysis/) [29]. Given the findings that the ALYREF gene region is amplified in breast cancer and ALYREF mRNA is upregulated in cancerous tissue, we further explored the relevance of intra-tumoral ALYREF mRNA expression level and its association with clinical outcome in patients. Using a breast cancer screening cohort (*n* = 128) (Table S1), we identified high levels of intra-tumoral ALYREF mRNA expression as significantly associated with poor disease-free survival (*p* = 0.041, log-rank test, Fig. 1D) and poor overall survival (*p* = 0.009, log-rank test, Fig. 1E). This negative prognostic impact of high ALYREF expression levels prevailed after adjustment for other well-known prognostic factors, including age, tumor stage, primary tumor size, grading, immunohistochemical subtype (estrogen, HER2 and progesterone receptor status) and Ki-67 proliferation index, using a multivariate Cox proportional model (Table S2) (hazard ratio (HR) and 95% confidence interval (CI) for disease-free survival: 2.72 (1.18–6.27), *p* = 0.0015, for overall survival: 3.2 (1.25–8.24), *p* = 0.018, respectively). Using a publicly available microarray-based large dataset (*n* = 1764 for DFS, *n* = 626 for OS) [28], high levels of ALYREF mRNA were independently validated to be a negative prognostic factor for disease-free (HR = 2.26 (1.9–2.69), *p* < 0.001, Fig. 1F) and overall survival (HR = 1.71 (1.16–2.51), *p* = 0.0059, Fig. 1G). As breast cancer is a heterogenous disease in terms of underlying biology, we analyzed different subtypes (by using the online tool bc-GenExMiner v4.7) and identified the highest ALYREF mRNA expression in the basal-like subtype (Fig. 1H, Table S3) and TNBC (Fig. S1B). As this subtype is associated with biological aggressiveness, we focused all of our following analysis on the TNBC subtype. As shown in Fig. 1I, high ALYREF mRNA expression was also associated with poor recurrence-free survival in pure TNBC patients (*n* = 320, HR = 1.99 (1.42–1.80), *p* < 0.0001). The explore ALYREF protein expression patterns in breast cancer, we found that the protein is strongly expressed in tumor samples of breast cancer patients (Fig. S1C), with the highest protein expression found in the basal subtype (Fig. S1D). Furthermore, ALYREF protein expression levels were evaluated in seven TNBC cell lines and a human mammary epithelial cell line (HMEC). The obtained results corroborate our findings, that ALYREF expression is increased in TNBC cell lines when compared to normal breast cells (Fig. S1E). Additional TMA tissue microarray IHC analysis of ALYREF expression demonstrates highly increased ALYREF expression in invasive breast carcinoma samples (*n* = 100) when compared to adjacent normal breast tissue (*n* = 10) (Fig. S1F). A previously published study showed higher ALYREF protein expression in tumor tissue compared to normal breast tissue [20] and using a publicly available cohort of breast cancer patients (*n* = 65), high ALYREF protein expression was associated with worse patient survival (Fig. S1G).

### ALYREF influences cellular growth, anchorage-independent growth and tumor sphere formation in triple-negative breast cancer

**Fig. 2:**
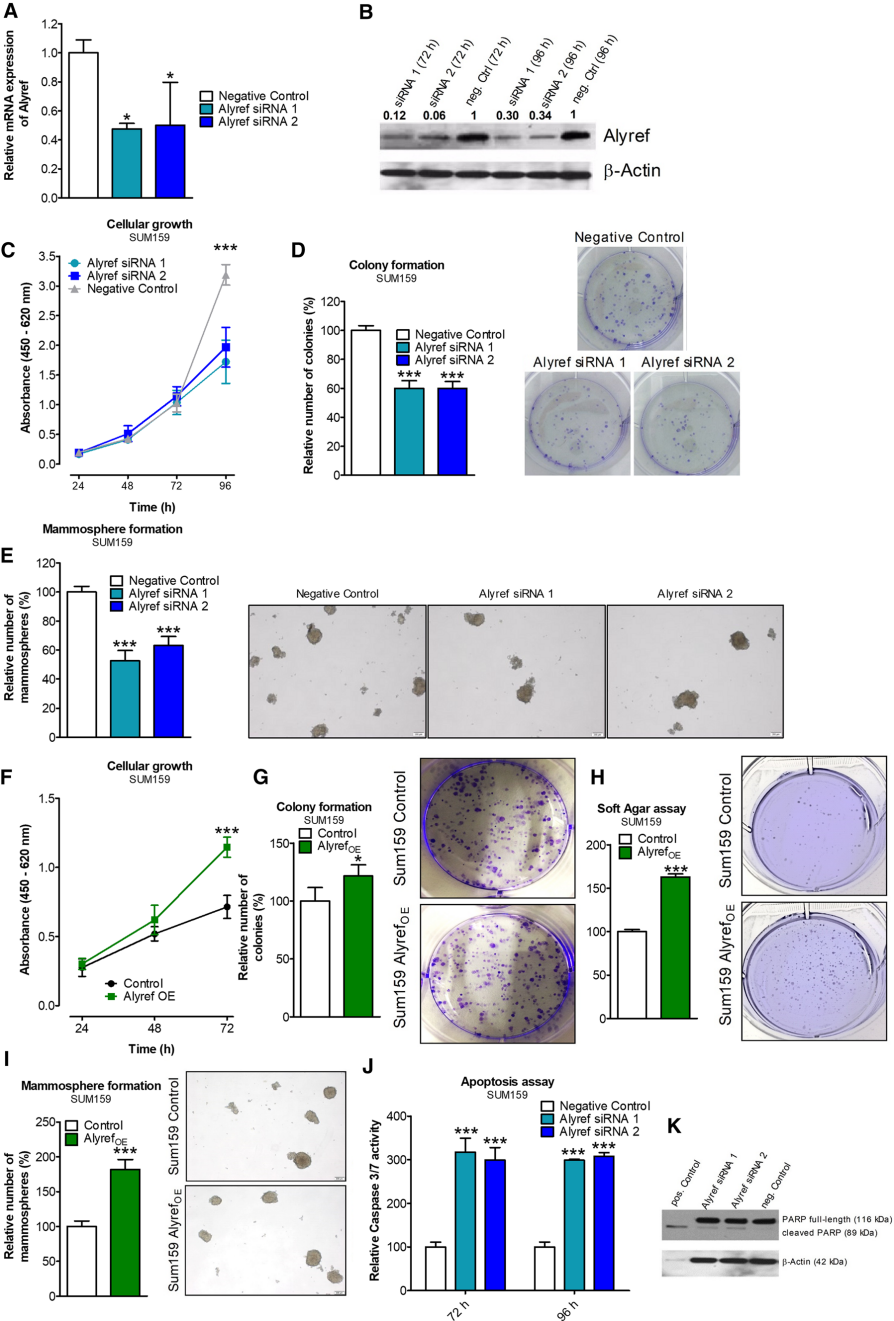
Cellular consequences of gain and loss of ALYREF function in triple-negative breast cancer cells. Verification of siRNA-mediated silencing efficiency of ALYREF in SUM159 cells on mRNA level (**A**) or protein level (**B**). Cellular growth assay in SUM159 cells over 96 h under control conditions (Negative Control siRNA; gray curve) or after siRNA-mediated knock-down of ALYREF (blue curves). *n* = 6, ± SD. ***p < 0.001. **D** Colony formation assay in SUM159 cells. Bar graphs on the left represent relative colony numbers under control conditions (Negative Control siRNA; white bars) or after siRNA-mediated ALYREF knock-down (blue bars). *n* = 3, ± SD. ***p < 0.001. Right panels depict corresponding representative pictures. **E** Mammosphere formation was analyzed either under control conditions (Negative Control siRNA; white bars) or after siRNA-mediated knock-down of ALYREF (blue bars) ten days after transfection. The relative numbers of spheres in ALYREF-silenced compared to control conditions are represented in the graphs. *n* = 3, ± SD. ***p < 0.001. Right panels depict representative pictures. **F** Cellular growth assay in SUM159 cells over 96 h under control conditions (black curve) or after overexpression of ALYREF (green curve). *n* = 6, ± SD. ***p < 0.001. **G** Colony formation assay in SUM159 cells under control conditions (white bars) or after ALYREF overexpression (green bar). *n* = 3, ± SD. *p < 0.05. Right panels depict corresponding representative pictures. **H** Soft agar assay in SUM159 cells under control conditions (white bars) or after ALYREF overexpression (green bar). *n* = 3, ± SD. ***p < 0.001. Right panels depict corresponding representative pictures. **I** Mammosphere formation was analyzed either under control conditions (white bar) or after overexpression of ALYREF (green bar) ten days after transfection. *n* = 3, ± SD. ***p < 0.001. Right panels depict representative pictures. **J** Caspase 3/7 assay either under control conditions (Negative Control siRNA, white bars) or after siRNA-mediated knock-down of ALYREF (blue bars) 72 and 96 h after transfection. *n* = 3, ± SD. ***p < 0.001. **K** Western Blot analysis of PARP shows the ratio of cleaved PARP to full-length PARP in ALYREF-silenced SUM159 cells 72 h after transfection. β-Actin was used as loading control and over-night staurosporine (1 µM) treatment as positive control. This figure contains data for SUM159 cells—data with the same experimental setup conducted in additional three TNBC cell lines can be found in the Supplementaries

Transient ALYREF knock-down (using two independent siRNAs, Fig. 2A, B; Fig S2, Fig. S3) in four independent TNBC cell lines (SUM159, MDA-MB-231, MDA-MB-468 and BT-549) significantly reduced cellular growth in all tested cell lines (Fig. 2C, Fig. S4A–C). To substantiate these findings with a second independent assay, we performed a colony formation assay and confirmed a significantly lower number of colonies in cells with decreased ALYREF expression levels (Fig. 2D, Fig. S4D–F).

We further investigated the influence of altered ALYREF expression on mammosphere formation and anchorage-independent colony formation in soft agar, both assays associated with stemness features of breast cancer cells. Under non-adherent FBS-free growth conditions, all four cell lines showed a decreased number and size of mammospheres in ALYREF-silenced cells (Fig. 2E, Fig. S4G–I).

In addition, TNBC cell lines (SUM159, the other two cell lines BT-549 and MDA-MB-468 were not able to form colonies in soft agar under the selected conditions) with decreased ALYREF expression formed significantly fewer colonies compared to control cells under anchorage-independent growth conditions (Fig. S5A, B). Conversely, after successfully generating SUM159 cells stably overexpressing lentivirus-transduced ALYREF (Fig. S6A, B), we observed more pronounced cellular growth, a higher number of colonies in the colony formation assay, increased colonies in the soft agar assay and increased mammosphere formation in ALYREF-overexpressing cells (Fig. 2F–I).

### ALYREF knock-down induces apoptosis and reduces mitochondrial energy metabolism in triple-negative breast cancer cell lines

**Fig. 3:**
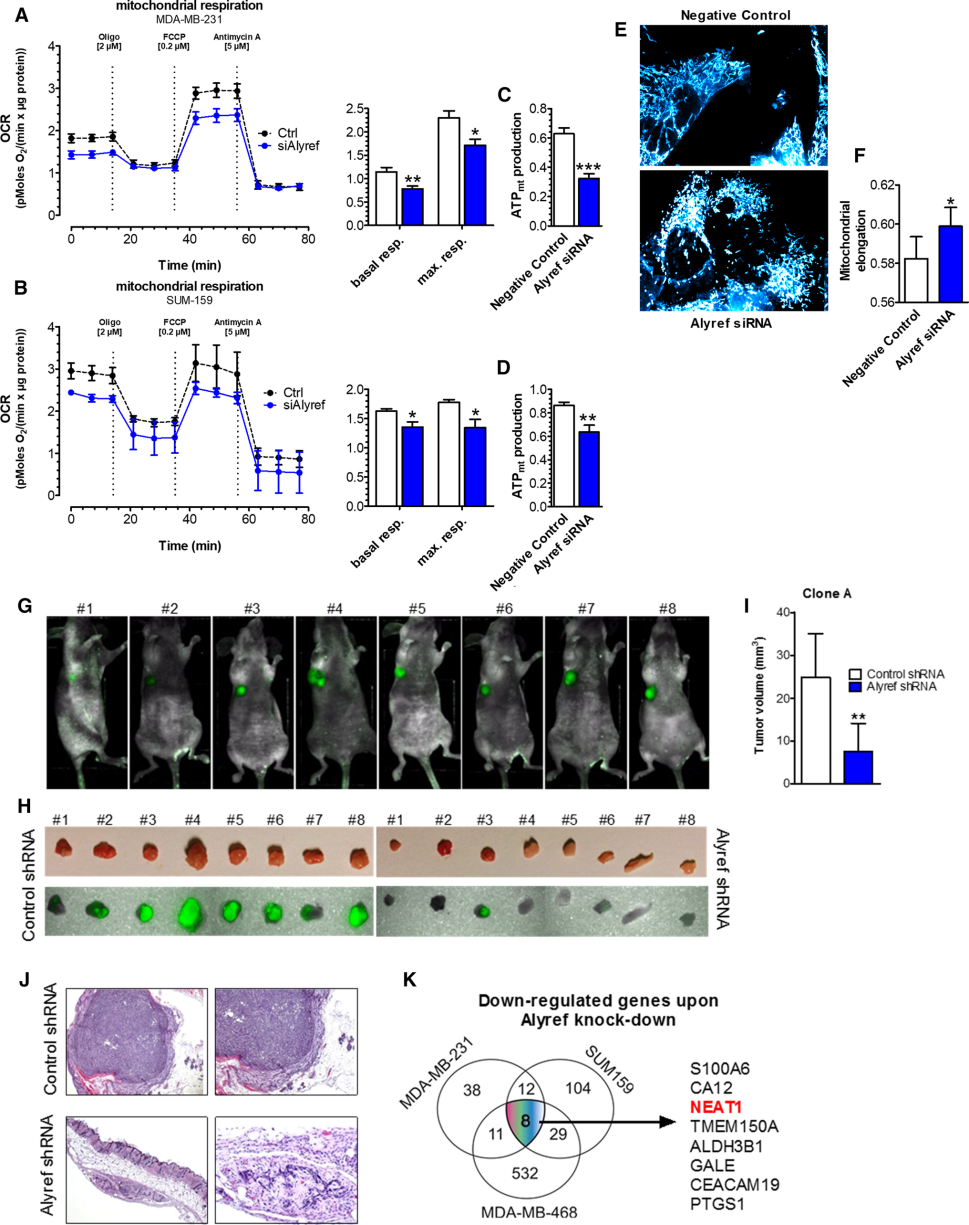
ALYREF silencing reduces mitochondrial metabolism and morphology and inhibits tumor growth in mouse xenograft tumors. **A**, **B** (left panels) Oxygen consumption rate (OCR) under control conditions (black curves) or after siRNA-mediated silencing of ALYREF (blue curve). OCR was normalized to protein content. As indicated, cells were treated with 2 µM oligomycin, 0.2 µM FCCP and 5 µM antimycin. (*Right panels*) corresponding statistical analysis of basal and maximal respiration, *n* = 3. **C**, **D** Corresponding statistical analysis of mitochondrial ATP production. Bars represent mean ± SEM. *p < 0.05, **p < 0.01, ***p < 0.001. **E** Representative pictures of mitochondrial morphology of SUM159 cells stained with mitoTracker® red FM for 30 min under control conditions (negative control siRNA; upper panel) or after siRNA-mediated silencing of ALYREF (lower panel). **F** Corresponding statistical analysis of (**E**) of mitochondrial elongation. *n* = 6, ± SD. *p < 0.05. **G**–**J** Mammary fad pad tumor formation of SUM159 cells transfected with tetracycline-inducible shRNA against ALYREF (clone A; left side) or control shRNA SUM159 cells (right side), *n* = 8. **G** Representative pictures of optical imaging of the whole body measuring the GFP intensity of xenograft tumors showing no fluorescence signal in tumor where ALYREF was silenced. **H** Corresponding optical imaging of the tumors after scarifying the mice on day 55. Upper panel shows macroscopically visible tumors and lower panel optical imaging of GFP signals of those tumors. **I** Bar charts showing a significantly decreased tumor volume from ALYREF-silenced cells, *n* = 8, ± SD. **p < 0.01. Data with the same experimental setup conducted with another clone (Clone B) can be found in Fig. S8*.* **I** Corresponding representative hematoxylin–eosin (H&E) stainings of tumors confirms no tumor formation in the ALYREF-silenced SUM159 cells. **K** Schematic picture of RNA-seq results depicting the eight common down-regulated genes upon ALYREF knock-down in three different cell lines (SUM159, MDA-MB-231 and MDA-MB-468)

After identifying ALYREF as an important factor in cellular growth, we aimed to clarify in more detail the mode of cellular action. Reduced levels of ALYREF led to a significant increase in caspase 3/7 activity after 72 and 96 h compared to control cells (Fig. 2J, Fig. S7A–C). In addition, Western blot analysis confirmed an increased cleavage of PARP (89 kDa band, a marker for increased apoptosis) in the TNBC cell lines (Fig. 2K, Fig. S7D–F). An increase in apoptotic cells was also detected using an AnnexinV FACS staining approach in SUM159 cells (Fig. S7G, H). To clarify whether the observed increase in apoptosis is due to alterations in energy metabolism, we performed measurements of key parameters of oxidative phosphorylation. Indeed, ALYREF knock-down led to decreased basal and maximal mitochondrial respiration (Fig. 3A, B) and reduced mitochondrial ATP production (Fig. 3C, D). Next, mitochondrial morphology was visualized on a confocal microscope, showing that while mitochondrial number and volume were not changed in ALYREF-silenced cells (Fig. S8A, B), mitochondrial morphology was significantly influenced. Knock-down of ALYREF resulted in a significant change of the mitochondrial elongation factor, showing that mitochondrial shape is more spherical (Fig. 3E, F), which is an indirect measure of reduced oxidative phosphorylation [30]. Together, these results suggest that ALYREF regulates cellular growth through regulation of apoptotic activity and influences mitochondrial energy metabolism.

### ALYREF expression levels influence tumor growth in vivo

To study the ALYREF effects in vivo, we established SUM159 cells with a tetracycline-inducible shRNA system against ALYREF mRNA. We established two independent shRNA clones (labeled clones A and B) in tetracycline-free conditions and verified reduced expression levels of ALYREF after doxycycline addition to the media on protein and mRNA levels (Fig. S9A, B). No significant difference in growth under tetracycline-free conditions compared to control cells was observed, indicating no differences in the endogenous growth characteristics of the ALYREF-inducible shRNA and control clones (Fig. S9C–E). However, inducing the shRNAs by adding doxycycline to the media corroborated our previous results by showing reduced cellular growth and colony formation in the shRNA-induced clones (Fig. S9F–H).

To confirm reduced tumor formation and a less aggressive phenotype in vivo, we evaluated primary tumor formation in nude mice after mammary fat pad injections of SUM159 cells carrying inducible shRNA and control clones. After 7 weeks, optical in vivo imaging clearly indicated a decrease/lack of green fluorescence signal in the tumors of ALYREF-silenced cells (Fig. 3G and Fig. S10A). After sacrificing the mice, macroscopic tumor volume measurements showed significantly smaller tumors formed by ALYREF-silenced cells, corroborating the histomorphometric analysis (Fig. 3H–J and Fig. S10B, C). Immunohistochemical analysis showed a strong nuclear ALYREF protein staining pattern in xenograft tumor samples of control tumors (Fig. S10D).

### Molecular mechanisms influenced by ALYREF in breast carcinogenesis

**Fig. 4:**
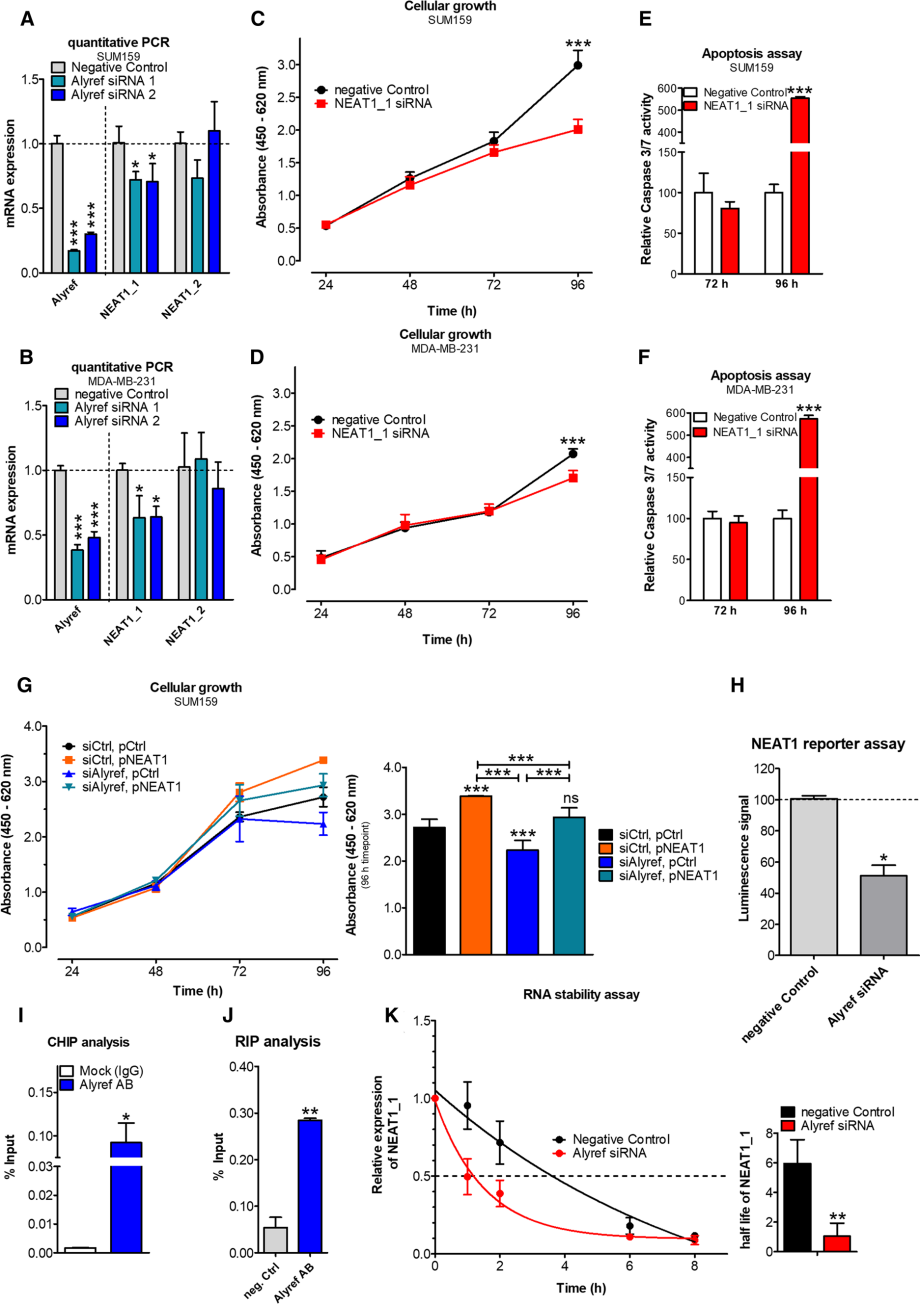
ALYREF binds to the promoter region, activates NEAT1 transcription and selectively regulates *the* stability of the short isoform of NEAT1. **A**, **B** Quantification of NEAT1_1 and NEAT1_2 (long isoform) expression levels after ALYREF silencing show a significant decrease only for the short NEAT isoform in two TNBC cell lines. *n* = 3, ± SD. **C**, **D** Cellular growth assay in these TNBC cells shows a decreased cellular growth upon NEAT1_1 knock-down, phenocopying the effect observed for ALYREF, *n* = 6, ± SD. ***p < 0.001. **E**, **F** Increased apoptosis activity measured by Caspase 3/7 assay upon NEAT1_1 knock-down. *n* = 3, ± SD. ***p < 0.001. **G** (Left panel) Rescue experiments in cellular growth assay in SUM159 cells show that by overexpression of the short NEAT1_1 isoform, the growth reducing effect upon ALYREF silencing could be reversed. For each condition negative control siRNA or an empty control plasmid have been used to allow for same conditions. (Right panel) Corresponding statistical analysis of the 96 h timepoint. *n* = 6, ± SD. ***p < 0.001. **H** NEAT1 promotor luciferase reporter assay in SUM159 cells either under control conditions (Negative Control siRNA) or after siRNA-mediated knock-down of ALYREF co-transfected with either an empty control plasmid pGL3 or pGL3 containing NEAT1 promotor elements. Bar graph represents the percentage of luciferase signal of cells treated with siRNA against ALYREF compared to control siRNA. *n* = 3, ± SD. *p < 0.05. **I** Chromatin immunoprecipitation in SUM159 cells with an antibody against ALYREF or a negative control antibody (Mock IgG) and subsequent qPCR using primers specific for NEAT1 promoter regions. Bars represent signal enrichment of selected regions adjusted to the 2% input sample when using the indicated antibodies of two independent IPs, ± SD. *p < 0.05. **J** RNA immunoprecipitation in SUM159 cells using an antibody against ALYREF or a negative control antibody shows a direct ALYRF::NEAT1_1 interaction. Bars represent data of qPCR with primers specific for NEAT1_1 isoform adjusted to the 10% input sample; ± SD. **p < 0.01. **K** RNA stability assay in SUM159 cells treated with 100 µg/ml actinomycin D either under control conditions (Negative control siRNA) or after ALYREF silencing. (Left panel) Graph representing NEAT1_1 decay over a period of 8 h. (Right panel) Corresponding calculated half-life of NEAT1_1 RNA

After identifying and establishing that ALYREF expression is relevant in human breast carcinogenesis and influences cellular growth and tumor formation in TNBC in vitro and in vivo, we performed RNA-seq whole transcriptome analysis to better understand the involved molecular mechanisms. By measuring changes in the whole transcriptome after ALYREF knock-down in three independent TNBC cell lines, we identified eight overlapping genes with a significant decrease in expression (of more than 50%), including S100 calcium binding protein A6 (S100A6), carbonic anhydrase 12 (CA12), nuclear enriched abundant transcript 1 (*NEAT1*), transmembrane protein 150A (TMEM150A), aldehyde dehydrogenase 3 family member B1 (ALDH3B1), UDP-glucose 4-epimerase (GALE), Carcinoembryonic Antigen Related Cell Adhesion Molecule 19 (CEACAM19), and Prostaglandin-Endoperoxide Synthase 1 (PTGS1) (Fig. 3K). As ALYREF has been previously proposed as an RNA-binding protein (8, 9) and the long noncoding RNA *NEAT1* has been involved in the growth and cancer stemness of TNBC [31], we decided to further decipher a possible link between these two molecules. As the *NEAT1* gene locus is transcribed into two overlapping isoforms, a short (*NEAT1_1*) and a long (*NEAT1_2*) form, we first confirmed the RNA-Seq data and differentiated the two isoforms by qRT-PCR using a pan-*NEAT1* (*NEAT1_1*) primer and a long isoform-specific *NEAT1_2* primer panel. Interestingly, only the short (pan) isoform of *NEAT1* (*NEAT1_1*) was significantly down-regulated in the ALYREF knock-down cells, whereas no difference in the long (*NEAT1_2*) isoform was detected (Fig. 4A, B). Conversely, and confirmatory, *NEAT1_1* expression was increased in stable ALYREF-overexpressing TNBC cells (Fig. S11A, B). To confirm that *NEAT1_1* silencing*,* but not *NEAT1_2* silencing, copies the observed ALYREF phenotype in TNBC cell lines, we established an isoform-specific siRNA-mediated *NEAT1* knock-down approach (Fig. S11C). *NEAT1_1* knock-down, but not *NEAT1_2* knock-down, resulted in the same phenotype observed in ALYREF knock-down cells, including reduced cellular growth (Fig. 4C, D; Fig. S11D, E) and increased apoptosis (Fig. 4E, F; Fig. S11F, G). Additionally, *NEAT1_1* silencing also reduced mitochondrial energy metabolism and ATP production (Fig. S12A–D) as well as changes in mitochondrial shape (Fig. S13A, B) but not mitochondrial count or volume (Fig. S14A, B). To further prove that the influence of ALYREF on the cellular growth pattern is mediated by the regulation of *NEAT1_1* expression, we performed a rescue experiment including transient overexpression of the short *NEAT1* isoform upon ALYREF knock-down. As shown in Fig. 4G, overexpression of the short isoform *NEAT1* resulted in increased cancer cell growth and rescued the ALYREF knock-down phenotype.

### ALYREF regulates *NEAT1* at transcriptional and selective the short isoform on the post-transcriptional levels

Since our data suggested a correlation between the expression and phenotype of ALYREF and the short isoform *NEAT1_1*, we further clarified whether ALYREF regulates the transcription of *NEAT1*. For this purpose, we used *NEAT1* promoter-containing luciferase reporter plasmid, where 2384 bp of the *NEAT1* promoter was integrated upstream of a firefly luciferase gene containing the pGL3 vector. Indeed, ALYREF silencing led to a significant reduction in luciferase activity in cells ectopically expressing the *NEAT1* promoter fragment (Fig. 4H) compared to control conditions. To further strengthen the link between ALYREF and NEAT1expression we investigated a possible contribution of ALYREF in NEAT1 expression. According to our findings above which postulated that ALYREF is regulating NEAT1 transcription at the promoter region, knock-down of ALYREF should result in decreased NEAT1 expression in the nucleus. To test this hypothesis, we applied fluorescence in situ hybridization (FISH) to visualize NEAT1 in the nucleus. SUM159 and MDA-MB-231 cells were either transfected with scrambled control siRNA or siRNAs against NEAT1 or ALYREF and incubated with NEAT1-specific FISH probes 48 h after transfection. Under both conditions (NEAT1 and ALYREF silencing), the total amount of NEAT1 signals decreased in the tested cell lines compared to control conditions (Fig. S15A–D). In contrast, in stable ALYREF-overexpressing SUM159 cells, the amount of NEAT signals increased compared to control cells (Fig. S15E, F). Taken together, these results support the hypothesis that ALYREF is controlling NEAT1 expression on the transcriptional level.

These results suggested that ALYREF directly or indirectly regulates *NEAT1* transcription in a region upstream of the core *NEAT1* promoter. To substantiate the results and demonstrate a direct interaction between the ALYREF protein and the *NEAT1* promoter sequence, we performed chromatin immunoprecipitation (ChIP) experiments. In accordance with the promoter construct assays, the ChIP procedure with primers covering the described regions of the *NEAT1* promoter (i.e., primers binding within 1200–1600 bp upstream of the *NEAT1* transcription start site) confirmed direct ALYREF binding to the *NEAT1* promoter within the first 2000 bp upstream from the transcription start site (Fig. 4I).

As ALYREF has been proposed as an RNA-binding protein, we further performed an RNA immunoprecipitation (RIP) assay that demonstrated a direct physical interaction between ALYREF and *NEAT1_1* but not *NEAT1_2* (Fig. 4J; Fig. S16). Continuative actinomycin D experiments further demonstrated that ALYREF prolonged the *NEAT1_1* half-life (Fig. 4K) but had no significant influence on the half-life of the long isoform *NEAT1_2* (Fig. S17). To further elucidate the underlying molecular mechanisms for the selectively transcriptional regulation of the short isoform of *NEAT1* by ALYREF action, we investigated the influence of ALYREF on *NEAT1* isoform-regulating CPSF6–NUDT21 (CFIm) complex.

**Fig. 5:**
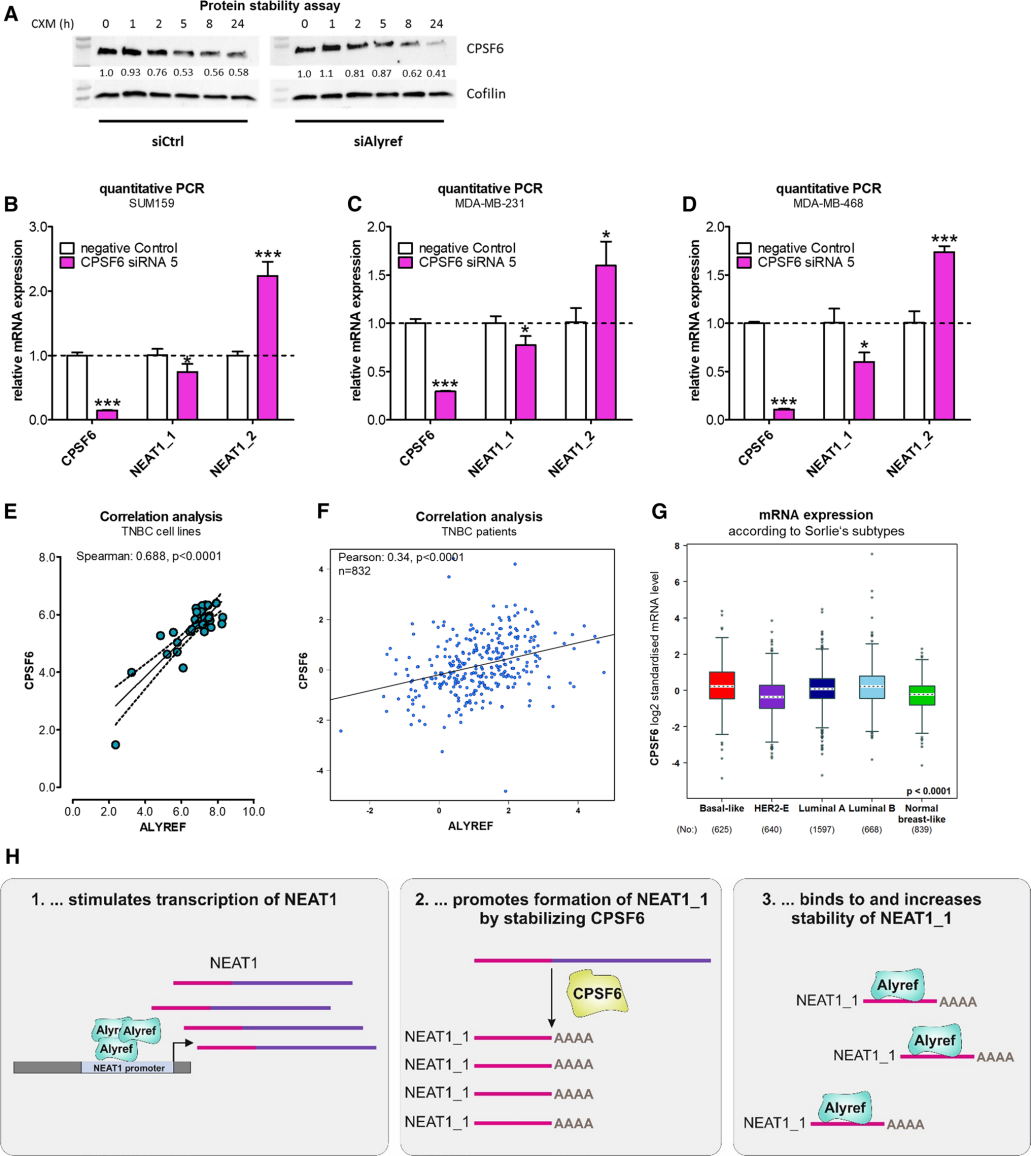
ALYREF stabilizes, correlates and regulates the NEAT1_1 activator CPSF6. **A** Cycloheximide chase assay to assess protein stability of CPSF6 under control conditions or after ALYREF silencing over 24 h. Cofilin was used as housekeeper. **B**–**D** Evaluation of RNA expression of CPSF6, NEAT1_1 and NEAT1_2 after siRNA-mediated CPSF6 silencing in three TNBC cell lines (*n* = 3, *p < 0.05, **p < 0.01, ***p < 0.001). **E** Correlation analysis of CPSF6 and ALYREF expression in TNBC cell lines. Data were derived from the publicly available depmap.org database (*n* = 30, Spearman correlation = 0.688, *p* < 0.0001). **F** Correlation analysis of CPSF6 and ALYREF expression in breast cancer patients restricted to TNBC. Data were derived from the publicly available bc-genexminer database (*n* = 832, Pearson correlation = 0.34, *p* < 0.0001). **G** RNA-sequencing data for mRNA expression of CPSF6 among breast cancer subtypes derived from bc-genexminer database. Specific *p* values are listed in Supplementary Table S4. **H** Schematic working hypothesis and model of ALYREF-mediated regulation of NEAT1 in triple-negative breast cancer

Overall, ALYREF did not influence CPSF6 or NUDT21 mRNA expression (Fig. S18A,B) or mRNA stability of those proteins (Fig. S19A, B). Interestingly, ALYREF stabilized CPSF6 protein levels (Fig. 5A, Fig. S20) but not NUDT21 stability (Fig. S21). Furthermore, as a proof of concept, knock-down of the *NEAT1_1* activator/*NEAT1_2* repressor CPSF6—efficiency tested on mRNA and protein level (Fig. S22A, B)—was decreasing *NEAT1_1* levels, whereas increasing the long isoform *NEAT1_2* RNA levels in three independent TNBC cell lines (Fig. 5B–D). No change in *NEAT1* isoform expression was detected after NUDT21 silencing (Fig. S23). To confirm the human relevance, CPSF6 expression was increased in breast cancer tissue (Fig. S24A; all subtypes), significantly correlated with ALYREF expression levels in breast cancer cell lines (Fig. S24B) as well as in breast cancer tissue samples (Fig. S24C), with the highest expression levels as well as strongest correlation in TNBC subtype (Fig. 5E–G, Table S4). There was no correlation between ALYREF and CPSF6 expression in the other subtypes (Fig. S24D–G). In summary and based on our findings, we propose ALYREF as a novel factor in TNBC tumorigenesis, through molecularly selective regulation of the short *NEAT1_1* isoform (Fig. 5H).

## Discussion

In this study, we identified and comprehensively characterized for the first time the role of the nuclear mRNA export factor ALYREF in triple-negative breast carcinogenesis. Overall, we found that the ALYREF gene is amplified in human cancers and that high expression levels are associated with poor clinical outcome in human breast cancer. A few previous studies described the expression of ALYREF in certain types of human cancer, including up-regulation in squamous cell carcinoma [32], ovarian cancer and lung cancer [20], whereas downregulation was described in skin and testicular cancers [20]. Importantly, there are no data so far published reporting the clinical relevance of ALYREF in breast cancer or TNBC. Our survival analyses of two independent breast cancer cohorts showed consistent results and suggest ALYREF as a novel prognostic biomarker that might be useful in the stratification of patients according to their individual risk. Based on this clinical observation, we further demonstrated a significant influence of ALYREF on the cellular growth of TNBC cells and tumor formation in vivo. Moreover, as we found that inducible knock-down of target ALYREF expression in orthotopic breast tumors led to significantly decreased tumor formation, these findings indicate the potential for therapeutic interventions to treat TNBC. Based on the human relevance and proliferative effects in cell lines and in vivo tumor formation in TNBC model systems, we aimed to unravel the mechanism behind ALYREF’s contribution to breast carcinogenesis.

In general, ALYREF is a heat stable, nuclear chaperone known to regulate dimerization, DNA binding and transcriptional activity of basic region-leucine zipper (bZIP) DNA binding domain-containing proteins [33] and mRNA export adaptors [13]. ALYREF regulates mRNA export by specifically binding to m^5^C-modified regions of mRNAs [15]. Based on our transcriptome analysis after ALYREF knock-down, several RNAs including the short isoform of the cancer-associated noncoding RNA *NEAT1*, were positively correlated (down-regulated) in the three tested TNBC cell lines.

*NEAT1* has previously been shown to be upregulated in several cancer entities, commonly exerting the function of a competing endogenous RNA (ceRNA) and possibly many other functions. The *NEAT1* gene is transcribed into two isoforms, *NEAT1_1* of 3.7 kb and *NEAT1_2* of 22.3 kb, where *NEAT1_1* completely overlaps with the 5′ end of *NEAT1_2. NEAT1_1* is the most abundant, oncogenic [34] isoform, whereas *NEAT1_2* seems to be an essential component for nuclear paraspeckle formation [35]. Recently, published studies also indicated that ALYREF can bind to and influence the function and nuclear export of other long noncoding RNAs in different cellular models [36, 37]. Furthermore, we considered the link between ALYREF and *NEAT1* as a potential interaction pair for a more comprehensive characterization since the short isoform of *NEAT1* seems to play an important role in breast carcinogenesis [31] based on the following points: (i)* NEAT1* is highly upregulated in breast cancer tissue of patients when compared to healthy surrounding tissue and is correlated with a higher TNM stage, increased occurrence of lymph node and distant metastases and a worse overall survival [38]. (ii)* NEAT1* expression is increased in breast cancer cell lines, and knock-down of *NEAT1* shows the same phenotype as ALYREF knock-down, i.e., *NEAT1* knock-down reduced cancer cell growth [15, 38], migration and invasion [15] as well as increased apoptosis [38] in vitro and reduced tumor size and metastasis formation in vivo [39, 40]. Since ALYREF and *NEAT1* were positively correlated in our model system, we hypothesized that ALYREF may regulate *NEAT1* at the transcriptional level. To substantiate our hypothesis, we performed *NEAT1* knock-down experiments showing that this lncRNA parallels the cellular changes and phenocopies ALYREF, giving more confidence to a functional link between this protein and lncRNA. Importantly, the observed influence of *NEAT1* on breast cancer cellular growth and apoptosis has been demonstrated by other groups in other breast cancer cell lines, underlining the generalizability of these findings [15, 38]. Furthermore, we showed that overexpression of the short isoform of *NEAT1* was able to rescue the ALYREF knock-down phenotype by reestablishing cellular growth to control condition levels. In addition to previously used cellular growth and apoptosis assays, we chose two alternative techniques connecting cellular apoptosis and cellular metabolism, i.e., studying mitochondrial function. Since these cell organelles are known contributors to cell metabolism as well as apoptotic processes [41, 42] and the main source for cellular ATP production [43], we assessed the influence of ALYREF and *NEAT1* on metabolic function. Measurement of the mitochondrial O_2_ consumption rate (OCR) directly represents oxidative phosphorylation (OXPHOS), and promoted OXPHOS has been demonstrated to increase cancer cell proliferation [44] and metastasis formation [45]. Therefore, we speculated that if ALYREF and *NEAT1* are essential for a balanced metabolic state of TNBC cells, silencing of either of them should lead to dysregulated mitochondrial respiration. Indeed, our results show that upon knock-down of ALYREF or *NEAT1,* basal and maximal mitochondrial respiration as well as mitochondrial ATP production were reduced compared to control conditions. Impaired mitochondrial ATP levels were previously demonstrated to be critical contributors to apoptosis execution [46]. Furthermore, we investigated mitochondrial morphology upon changing the expression levels of ALYREF and *NEAT1*. Due to their numerous cellular functions, mitochondria are characterized by enormous morphological plasticity [47]. Changes in mitochondrial morphology occur not only upon apoptosis induction, mitochondrial shifting from reticulotubular to punctiform [48] and more spherical shape [49] but also upon alterations in cellular metabolism. Several groups have demonstrated impaired mitochondrial fusion (i.e., more spherical mitochondrial shape) decreases oxidative phosphorylation, thereby connecting cellular metabolism with mitochondrial shape [30]. In our study, the total mitochondrial count and mitochondrial volume per cell were unchanged upon knock-down of ALYREF and *NEAT1*, but marked alterations of the mitochondrial elongation factor were observed, which indicated a more spherical morphology in ALYREF- and *NEAT1*-silenced cells than in control cells. These data support our findings that ALYREF and *NEAT1* are important for breast cancer survival and that downregulation results in apoptosis induction, which is in line with recent findings of Wang et al. [50], who showed that *NEAT1* depletion affects mitochondrial structure and function. Our mechanistic studies decipher a dual role for ALYREF in the regulation of the *NEAT1_1* isoform, where both transcriptional and post-transcriptional mechanisms through direct promoter and RNA-binding features are involved. The findings that ALYREF exclusively regulates the RNA levels of *NEAT1_1* seem surprising at first sight, since both *NEAT1* isoforms share the same promoter and transcriptional activation should affect both, *NEAT1_1* and *NEAT1_2*. *NEAT1_1* transcripts are generated by canonical 3’-processing via the CPSF6-NUDT21 (CFIm) complex and are cleaved at the polyadenylation signal located upstream. The CFIm components Cleavage And Polyadenylation Specific Factor 6 (CPSF6) [51] and Nudix Hydrolase 21 (NUDT21), therefore, are referred to as *NEAT1_1* isoform activators [52]. By stabilizing CPSF6 protein stability, ALYREF is therefore shifting the post-transcriptional processing dynamics towards *NEAT1_1* isoform generation, explaining the exclusive effect on the short *NEAT1* isoform. Our study is not answering whether targeting ALYREF in normal cells or normal breast epithelial, will result in the same effects when targeting breast cancer cells. However, the significantly higher expression especially in basal-like breast cancer might open a therapeutic window to use ALYREF as a therapeutic molecule. We identified ALYREF as transcriptional and post-transcriptional regulator involved in breast carcinogenesis but our findings do not exclude the other cellular functions of ALYREF as potential contributors to carcinogenesis such as its mRNA export function [15] or its role in maintaining genomic stability [19]—therefore, additional studies of ALYREF function are warranted to get a more detailed picture of this obviously important molecule.

## Conclusions

Overall, our data describe for the first time a crucial contribution of ALYREF to TNBC carcinogenesis. This work establishes ALYREF as a potential prognostic factor in TNBC and demonstrates that ALYREF is important in breast tumor formation, at least in part, through the transcriptional and post-transcriptional regulation of the short *NEAT1* isoform. Further studies and clinical trials are warranted to fully determine the potential role of ALYREF as a novel therapeutic target for patients with TNBC.

### Supplementary Information

Below is the link to the electronic supplementary material.

Supplementary file1 (DOCX 39 KB)

Supplementary file2 (DOCX 21 KB)

Supplementary file3 (PPTX 23516 KB)

Supplementary file4 (DOCX 41 KB)

## Data Availability

All data supporting the findings of this study are available from the corresponding author upon request.
