# Supplementary material for: ALYREF, a novel factor involved in breast carcinogenesis, acts through transcriptional and post-transcriptional mechanisms selectively regulating the short NEAT1 isoform
Source: Cell Mol Life Sci. 2022 Jul 1;79(7):391. doi: 10.1007/s00018-022-04402-2 (PMC9249705; doi:10.1007/s00018-022-04402-2)
Supplement: Supplementary file 1 — Supplementary file1 (DOCX 39 KB) [file 18_2022_4402_MOESM1_ESM.docx]

**Supplementary Material and Methods**

Cell Culture

The breast cancer lines MDA-MB-231, MDA-MB-468, BT-549 and human mammary epithelial cells (HMEC) were purchased from American Type Culture Collection (ATCC, Manassas, Virginia, USA), the cell line SUM159 was obtained from Asterand (Detroid, MI, USA). SUM159 (RRID:CVCL_5423) cells were maintained in Ham`s F12 containing 1 mM L-Glutamine (GE Life Sciences, Chicago, Illinois, USA), 2 mM HEPES buffer (Gibco, Darmstadt, Germany), 5 µg/ml insulin actrapid (Novo Nordisk, Vienna, Austria), 1 µg/ml hydrocortisone (Sigma-Aldrich, Vienna, Austria), 1% penicillin/streptomycin (Sigma-Aldrich) and 5% FBS (Biochrom, Berlin, Germany). MDA-MB-231 (ATCC Cat# HTB-26) and MDA-MB-468 (ATCC Cat# HTB-132) were grown in DMEM (high glucose [4.5 g/L], Gibco, Carlsbad, CA, USA), 10% FBS (Biochrom) and 1% penicillin/streptomycin (Sigma-Aldrich). BT-549 (ATCC Cat# HTB-122) were maintained in RPMI 1640 (with L-Glutamine, Gibco) 10% FBS (Biochrom) and 1% penicillin/streptomycin. HMEC cells were cultivated in Mammary Epithelial Cell Basal Medium (ATCC; PCS-600-030) supplemented with Mammary Epithelial Cell Growth Kit (ATCC; PCS-600-040). Cell line authentication was performed via STR-analysis.

All cell lines were kept at 37°C in a humidified 5% CO_2_ atmosphere. After obtaining a confluence of approximately 70%, total RNA was isolated following a standard Trizol (Invitrogen, Carlsbad, CA, USA) protocol according to the manufacturer’s instructions and RNA was stored at -80°C until further procedures.

TMA Tissue Microarray

In order to compare ALYREF expression levels in tumor versus healthy breast tissue, we performed a tissue microarray immunohistochemistry experiment. An invasive breast cancer tissue microarray with 100 invasive breast cancers and 10 samples of normal breast tissue was purchased from AMS Biotechnology, Abingdon, UK, Europe (cat.: BC081120f). The microarray comprised hormone-receptor positive, triple negative and HER2 positive breast cancers. We used a moncolonal mouse antibody for ALYREF (clone 11G5, cat.: ab6141, Abcam, Cambridge, UK, Europe) at a dilution of 1:100. Immunohistochemical staining was performed on a Ventana Benchmark Ultra stainer, (Roche Diagnostics, Vienna, Austria, Europe) using the following settings: pretreatment at pH 6.0 (CC2 setting), 32 min incubation time, visualization by Ventana ultraView DAB. Overall staining intensity (cytoplasmic and nuclear) was assessed semi-quantitatively by a board approved pathologist (S.J.) on a 4 tiered scale of 0-3 with 0 denoting no staining and 3 corresponding to strong tumor cell staining.

Transient Transfection with siRNAs and overexpression plasmids

To reduce expression of our target genes, siRNA-mediated knock-down according to the fast-forward protocol (Qiagen) was performed. Plasmid transfection was performed with Lipofectamine® 2000 Transfection Reagent (Thermo Fisher Scientific) according to manufacturer’s protocol. For ALYREF two different siRNAs were used (Hs_THOC4_5 FlexiTube siRNA, Hs_THOC4_6 FlexiTube siRNA; Qiagen, Hilden Germany), for NEAT1_1 Hs_*NEAT1*_3 FlexiTube siRNA (Qiagen) and for NEAT1_2 an siRNA with the sequence 5’-CAA ACU CUG UAC CCA UUA ATT-3’ (Microsynth, Balgach, Switzerland) were used. AllStars Negative Control (Qiagen) and AllStars Cell Death Control (Qiagen) were used as controls. For the NEAT1 rescue assay the plasmid pCRII_TOPO_hNEAT1 was used (this plasmid was a gift from Archa Fox (Addgene plasmid # 61518; http://n2t.net/addgene:61518; RRID:Addgene_61518)) (1). Throughout the whole manuscript NEAT1_1 refers to the pan-isoform of NEAT1 since NEAT1_1 and NEAT1_2 are identical only that NEAT1_2 is the extended version.

Inducible shRNA lentiviral particles transduction (Tet-on system) & stable ALYREF overexpression

SUM159 cells were seeded 24 hours prior to lentiviral infection in complete growth medium. At the time of transfection, medium was changed in complete growth medium with Tet-free FBS. Cells were infected by adding 50 µl TetR (AmsBio, Abingdon, U.K.) lentiviral particles, 8 µg/ml polybrene and 10 µl of ViralPlus Transduction Enhancer. Stably transduced cells were selected using 1 µg/ml puromycin dihydrochloride. Afterwards cells were transfected once more using 10 µl inducible ALYREF shRNA lentiviral particles with GFP (AmsBio) or non-silencing control lentiviral particles, 8 µg/ml polybrene and 10 µl of ViralPlus Transduction Enhancer. The shRNA lentiviral particles target the same sequence, as ALYREF siRNA6 (Hs_THOC4_6 FlexiTube siRNA, Qiagen). GFP expressing cells were sorted using a fluorescence-activated cell sorter (FACS) and selected using 1 µg/ml puromycin dihydrochloride to obtain different clones of stable transduced cells. Cells were kept under Tet-free conditions to avoid induction of the shRNA. To induce the shRNA targeting ALYREF, 1 µg/ml doxycycline was added.

For stable ALYREF overexpression SUM159 and MDA-MB-231 cells were seeded and incubated overnight in complete growth medium. On the day of transfection, the medium was replaced with complete growth medium containing 8 µg/ml polybrene (Santa Cruz Biotechnology, Santa Cruz, CA, USA) and ViralPlus Transduction Enhancer (1:200, ABM, Richmond, BC, Canada). Cells were infected by adding 10 µl of human ALYREF lentiviral particles (ABM) or blank control lentiviral particles (ABM), respectively. Stably transfected cells were selected with 1 µg/ml puromycin dihydrochloride (Gibco, Carlsbad, CA, USA).

Quantitative RT-PCR

Quantitative RT-PCR was carried out in technical duplicates of biological triplicates using primers specific for ALYREF and *NEAT1* (Eurofins Genomics, Vienna, Austria). Primer sequences are the following:

| **Gene** | **forward** | **reverse** |
| --- | --- | --- |
| ALYREF | CATGAAGCAGTACAACGGCG | CTGTTTACGCTCTGTGCAGG |
| NEAT1_1 | CTTCCTCCCTTTAACTTATCCATT | CTCTTCCTCCACCATTACCAACAATAC |
| NEAT1_2 | CAGTTAGTTTATCAGTTCTCCCATCCA | GTTGTTGTCGTCACCTTTCAACTCT |
| CPSF6 | TGGTTCTGGATCAAGACGTGAA | TGACGGTCTCTACTACGGGA |
| NUDT21 | TGTACATGAGCACCGGCTAC | TCCTGACGACCCAGTATCTCT |
| GAPDH | AAGGTCGGAGTCAACGGATTT | ACCAGAGTTAAAAGCAGCCCT |
| B2M | TGCTGTCTCCATGTTTGATGTATCT | TCTCTGCTCCCCACCTCTAAGT |
| U6 | CTCGCTTCGGCAGCACA | AACGCTTCACGAATTTGCGT |

Quantitative RT-PCR was done on a LightCycler® 480 Real-Time PCR System (Roche Diagnostics, Mannheim, Germany) using the QuantiTect SYBR Green PCR Kit (Qiagen) according to the manufacturer’s standard protocol. The arithmetic mean of the housekeeping genes GAPDH and B2M or U6 in case of lncRNA detection was used for normalization and relative gene expression levels were calculated using a standard 2^-ΔΔCT^ method (2).

Cellular Growth Assays

Cell proliferation was assessed by the WST-1 proliferation assay (Roche Applied Science, Vienna, Austria). Therefore, cells were incubated for 24-96 hours and every 24 hours the WST-1 proliferation reagent was applied according to the manufacturer’s recommendations. The colorimetric changes were measured ever day using a SpectraMax Plus (Molecular Devices, San José, CA, USA) at a wavelength of 450 nm with a reference wavelength at 620 nm. Three independent experiments were performed.

For colony formation assay, cells were trypsinized 24-48 hours after transient transfection. Inducible cells were induced with 1 µg/ml doxycycline (Clontech). After trypsinization cells were counted and seeded for colony formation assay in 6-well plates at a density of 100-400 cells/well (depending on the cell line). After 10-14 days, cells were fixed and stained with crystal violet and the number of colonies was counted. A colony is defined to consist of at least 50 cells. Each experiment was carried out in biological and technical triplicates.

Caspase 3/7 Assay

Activity of caspases 3 and 7 was determined by Caspase-Glo 3/7 assay (Promega, Madison, WI, USA) according to the manufacturer’s instructions. After adding the substrate, luminescence was recorded using a luminometer (LumiStar, BMG Labtech, Ortenberg, Germany) 72 and 96 hours after transient transfection. Stable transduced cells were also seeded in 96-well plates and ALYREF shRNA was induced using 1 µg/ml doxycycline (Clontech).

Protein extraction and Western Blot analysis

Total protein was extracted with radio-immunoprecipitation assay (RIPA) buffer (Biorad, Hercules, CA, USA). 25 µg of total cellular proteins were resuspended in laemmli buffer (Biorad) and heated at 95 °C for 5 minutes. Proteins were separated by a 4–15% Mini-PROTEAN® TGX™ Precast Gel (Biorad) and transferred onto a nitrocellulose membrane (Applichem, St. Louis, MO, USA). The membrane was blocked for 1 hour with 5% non-fat dry milk in Tris buffered Saline/0.1% Tween-20. Immunoblotting was performed and antibodies specific for ALYREF (THOC4/Aly, Cell Signaling Technology Cat# 12655, RRID:AB_2797980 Danvers, MA, USA), the apoptosis marker PARP (Cell Signaling Technology Cat# 9542, RRID:AB_2160739), CPSF6 (Cell Signaling Technology Cat# 75168, RRID:AB_2876852), NUDT21 (Abcam Cat# ab183660, RRID:AB_2827670), Cofilin (Abcam Cat# ab42824, RRID:AB_879739) and β-Actin (Sigma-Aldrich Cat# A5441, RRID:AB_476744) were detected using HRP-conjugated anti-mouse (Agilent Cat# P0161, RRID:AB_2687969) or anti-rabbit (Agilent Cat# P0448, RRID:AB_2617138) antibodies, respectively (Agilent/Dako, Glostrup, Denmark). Visualization was performed using an enhanced chemoluminescence detection system (Super Signal West Pico, Thermo Scientific, Waltham, MA, USA). As positive control for apoptosis induction cells were treated with 1 µM staurosporine (Sigma-Aldrich) overnight.

Flow cytometric Annexin V assay

To determine the percentage of apoptotic cells, a flow cytometric assay was performed employing a dual Annexin V and Propidium iodide (PI) staining. For this purpose, SUM159 cells were seeded in 6 cm dishes (1.5 x 10^5^ cells/dish) and were transfected with 20 nM of the respective siRNAs using HiPerfect (Qiagen) according to the fast-forward protocol. After 72 h, supernatant was collected and cells were harvested by trypsinization. Cell pellets were first washed with PBS, then with Annexin V buffer (100 mM HEPES, 1.4 M NaCl, 25 mM CaCl_2_). Subsequently, cells were stained in Annexin V buffer containing 1 μg/ml Propidium iodide (Sigma-Aldrich) and APC-Annexin V (BioLegend, San Diego, CA, USA) in a 1:80 dilution. After 15 min incubation, cells were measured on a CytoFLEX SI (Beckman Coulter) recording 20 000 events per sample. The number of early apoptotic cells (gated for Annexin V positive) and the number of late apoptotic cells (gated for Annexin V and PI double-positive) were summed up to receive the percentage of apoptotic cells for each sample.

Anchorage-independent growth assay

The efficiency of colony formation of transfected cells in soft agar was determined by plating 2,500 cells in 1 ml of complete growth medium containing 0.35% low gelling temperature agarose (Sigma-Aldrich) topped over 1.5 ml of growth medium containing 0.5% agar (Sigma-Aldrich) in a 35mm dish. Cells were cultured at 37°C and 5% CO_2_ for up to 4 weeks. Colonies were stained with 0.005% crystal violet (Sigma-Aldrich) in 20% methanol and the number of colonies was counted using a dissecting microscope.

Mammosphere formation assay

To assess the effect of low ALYREF expression on the self-renewal capacity (mammosphere formation), we performed a spheroid growth model as previously described (3) with slight modifications. In detail, the adherent growing breast cancer cell lines were dissociated into single cells using trypsin/EDTA and 2,000 single cells per well were seeded in ultra-low attachment 6-well plates (Corning, NY, USA) using serum-free MEBM (Lonza, Basel, Switzerland) medium (SFM). SFM was supplemented with 1xB27 supplement (Gibco), 20 ng/ml human epidermal growth factor EGF (Peprotech, Hamburg, Germany), 10 ng/ml human basic fibroblast growth factor FGF (Peprotech), 20 IU/ml Heparin (Baxter, Vienna, Austria) and 1% antibiotic/antimycotic solution (Sigma-Aldrich). Mammospheres were observed and counted under a microscope 10 days later. Three independent experiments per cell line with each three technical replicates were performed.

Xenograft experiments

For tumor xenograft experiments, female, five week-old nude mice were obtained from Charles River Breeding Laboratories (Sulzfeld, Germany). Stably transfected inducible shRNA ALYREF-silenced (clone A and B) SUM159 or control shRNA SUM159 cells were re-suspended in phosphate-buffered saline (PBS, 1:1 mixed with matrigel) and subcutaneously injected at a density of 1×10^6^ cells into the mammary fat pad of the mice. Cells with ALYREF shRNA were injected in the left mammary fad pad, shRNA control cells in the right. Eight mice received clone A, six mice clone B. We tested two different clones (A and B) with the same ALYREF shRNA (equivalent to shRNA 6 in the transient transfection experiments). Doxycycline (Clontech) was administered through their drinking water in a concentration of 200 µg/mL. Tumor burden was assessed every few days by caliper measurements.

At day 55, tumor volume was estimated by using caliper measurements and all tumors were harvested for histologic analysis. Tumor volume and histologic stains were compared between mice with ALYREF shRNA and shRNA Control. All animal work was done in accordance with a protocol approved by the Institutional Animal Care and Use Committee at the Austrian Federal Ministry for Science and Research (BMWF) (BMWFW-66.010-0046-WF-V-3b-2016).

RNA Sequencing

The cDNA libraries were prepared using the TruSeq® Stranded Total RNA kit (Illumina Inc., San Diego, CA) according to the manufacturer´s recommendation. Briefly, 1µg of total RNA was used for first-strand synthesis performed with a random hexamer and SuperScript II (Life Technologies, Carlsbad, CA, USA). Second-strand synthesis was performed using dUTP and the Illumina-specific Second Strand Marking Master Mix. After end repair and A-tailing indexed adaptors were ligated to the cDNA fragments. Fragments successfully ligated with adaptor molecules on both ends were enriched by PCR for 15 cycles and purified with AMPure XP Beads (Beckman Coulter Inc., Brea, CA). The final libraries were quality checked on an Agilent Bioanalyzer and quantified with qPCR using a commercially available PhiX-library (Illumina Inc., San Diego, CA) as a reference. All samples were run in biological triplicates. A total of 6 equimolarily pooled samples were sequenced on Mid Output flow cells on an Illumina NextSeq in a paired end run with 2x 75 cycles. Raw RNA-Seq reads were aligned to the human hg19 genome using STAR (4). Gene read counts are generated using feature Counts (5) and differential expression was analyzed using DESeq2(6). Genes with adjusted p-value lower than 0.05 were considered differentially expressed. Upregulated and downregulated genes of the three cell lines were intersected.

Mitochondrial respiration measurements

SUM159, MDA-MB-231 and MDA-MB-468 cells, treated with either negative control siRNA or siRNAs against ALYREF or *NEAT1*, were plated on 6-well plates 72 hours prior to measurement. 24 hours before measurement cells were plated in XF96 polystyrene cell culture microplates (Seahorse Bioscience®, Agilent; CA, USA) at a density of 40.000 cells/well for SUM159 and 50.000 cells/well for MDA-MB231 and MDA-MB-468. After overnight incubation, cells were washed with unbuffered XF assay medium supplemented with 1 mM sodium pyruvate and kept in a 37°C and non-CO_2_ incubator for 40 minutes. Basal oxygen consumption rate (OCR) was measured using an XF96 extracellular flux analyzer (Seahorse Bioscience). A standard protocol was set up consisting of 15 minutes basal measurement followed by 2 µM oligomycin, addition of 0.2 µM FCCP and 5 µM antimycin A. Oxygen consumption was normalized to protein content (pmol O_2_/min x µg protein).

Confocal imaging and analysis of mitochondrial morphology

To monitor the influence of ALYREF or *NEAT1* silencing on mitochondrial morphology, SUM159 cells were plated on 30 mm glass cover slips in 6-well plates and transiently transfected with either Allstar negative control siRNA or siRNAs specific against ALYREF or *NEAT1*. 48 – 72 hours after transfection, cells were washed twice with EHL buffer (135 mM NaCl, 5 mM KCl, 2 mM CaCl_2_, 1 mM MgCl_2_, 10 mM Hepes, 2.6 mM NaHCO_3_, 440 µM KH_2_PO_4_, 340 µM Na_2_HPO_4_, 10 mM D-glucose, 0.1% vitamins, 0.2% essential amino acids, and 1% penicillin-streptomycin, pH adjusted to 7.4) and stained with mitotracker red FM (Thermo Fisher; 1:2000 – 0.5 µM) for 30 minutes, followed by three times washing with EHL buffer. Mitochondrial morphology was recorded by using a confocal spinning disk microscope (Axio Observer.Z1 from Zeiss, Jena, Germany) equipped with a 100x objective (Plan-Fluor x 100/1.45 Oil, Zeiss) and a Nipkow-based confocal scanning unit (CSU-X1, Yokogawa Electric Corporation, Tokyo, Japan). Z-stacks of mitochondria with 0.2 µm increments were imaged. The image stacks were deconvoluted using a blind deconvolution algorithm (NIS-Elements, Nikon, Austria). Morphology parameters were measured automatically with a costume made ImageJ macro using the following procedure. A background subtraction using the rolling ball method was used to further increase contrast for later analysis. Both, a global auto Otsu threshold using the stack histogram as well as a local auto Otsu threshold using the single slice histogram were applied to the stack and merged. The elongation factor was defined as the ratio of the small divided through the large diameter of the ellipsoid fitting. The ImageJ plugin 3D manager was used to segment the binarized mitochondria. With the plugin 3D Geometrical Measure the mitochondrial volume was determined. The Plugin 3D Ellipsoid Fitting generated an ellipsoid fit of the mitochondria to measure elongation parameters. For better visualization pictures were colored after imaging with ImageJ Software in hot-cyan.

*NEAT1* luciferase reporter assay

To test whether ALYREF silencing affects *NEAT1* transcription, we performed a *NEAT1* signal reporter assay according to standard procedures and as described by Lellahi et al. (7). The empty reporter plasmid encodes for firefly luciferase (pGL3 plasmid, Promega). To test if ALYREF silencing influences *NEAT1* transcription, the promoter region of *NEAT1* was cloned into the empty vector upstream of the luciferase sequence. To determine the region of ALYREF binding to the promoter, three plasmids were used, which contain different lengths of the *NEAT1* promoter i.e. 470 bp, 2384 bp and 4040 bp as described in Lellahi et al (7). In detail, 4x10^4^ SUM159 or 6x10^4^ MDA-MB-231 and MDA-MB-468 cells were seeded in complete growth medium in 24-well plates and transfected either with 20 nM Allstar negative control siRNA or 20 nM siRNA against ALYREF using the Qiagen fast forward transfection protocol and HiPerfect transfection reagent. After 24 hours, cells were transfected with either the empty pGL3 vector or the *NEAT1* promoter constructs together with the Renilla control plasmid (for normalization of luciferase activity) using lipofectamine 2000 reagent. After 24 hours, cells were lysed in 100 µl of passive lysis buffer according to the Dual Luciferase Reporter Assay System (Promega; 20 µl of the lysate were used for the luciferase activity measurements following the manufacturer instructions. Luciferase assays were run on a LUMIstar Luminometer (BMG Labtech, Ortenberg, Germany) in three independent biological replicates.

FISH (Fluorescence in-situ hybridization)

Cells were plated on 18 mm cover-slips (VWR, Vienna, Austria) in 6 well-plates and transfected with siRNAs (AllStar Negative Control siRNA, ALYREF siRNA1/5 and NEAT1 siRNA) as described above. 48 hours after transfection cells were treated with commercially available NEAT1 and GAPDH probes (Stellaris® FISH probes against human NEAT1 5’ segment #SMF-2036-1 and human GAPDH #SMF-2026-1; Biosearch Technologies, Petulama, CA, USA) according to manufacturer’s protocol. Cover-slips were placed on microscope slides with ProLong® Gold Antifade Mountant containing the nuclear dye DAPI (ThermoFisher Scientific). Images were generated with a confocal spinning disk microscope (Axio Observer.Z1 from Zeiss) equipped with a 100x objective lens (Plan-Fluor x 100/1.45 Oil, Zeiss) and a Nipkow-based confocal scanning unit (CSU-X1, Yokogawa Electric Corporation). Z-stacks of cells with 0.2 µm increments were imaged. Paraspeckle quantification (total amount of paraspeckles per DAPI-visualized cell) was performed manually by displaying the maximal intensity projection of the individual Z-stacks with the software NIS-Elements Viewer 4.20 (Nikon, Tokyo, Japan) allowing the quantification of all paraspeckles present in the respective cell.

RNA immunoprecipitation

To investigate if ALYREF and *NEAT1* directly interact, we performed an RNA Immunoprecipitation (Magna RIP^TM^, Millipore, Burlington, USA). Therefore, SUM159 cells were seeded on T-225 flasks until they reached 90% confluency. RIP was conducted according to manufacturer’s protocol. Each RIP consisted of three reactions using 5 µg of the following antibodies i.e. ALYREF (THOC4/ALY (D3R4R) Rabbit mAb, Cell Signaling) for the target sample, p54nrb/NONO as positive control – proven interaction of p54nrb/NONO and *NEAT1* (8) (Anti-p54nrb/NONO, clone 78-1-C6, Millipore) and rabbit or mouse IgG as negative control (same species as target antibody, included in the RIP kit). Input as well as IP-samples were analyzed with Western Blot, qRT-PCR and standard PCR.

Chromatin Immunoprecipitation (ChIP)

ChIP analysis of SUM159 cells was performed using SimpleChIP® Enzymatic Chromatin IP Kit (Magnetic Beads) (Cell Signaling) according to manufacturer’s protocol. Crosslinking of proteins and DNA was performed by incubating cells in formaldehyde (final concentration of 1%). DNA was sheared to fragments of 150 – 900 bp by sonication (Bioruptor, Diagenode, Seraing, Belgium). The samples were subsequently immunoprecipitated over night at 4°C using 2 µg of an antibody against ALYREF (i.e. THOC4/Aly, Cell Signaling Technology Cat# 12655, RRID:AB_2797980) or rabbit Normal IgG antibody as corresponding negative control or Histone H3 (D2B12) XP® rabbit mAB as positive control supplied with the kit. After chromatin purification samples were analyzed with qRT-PCR using specific ChIP primers for *NEAT1* followed by analysis of PCR products using a 2% agarose gel.

Immunohistochemical staining of ALYREF

IHC of 5 µm sections of paraffin-embedded speciment was performed using rabbit anti-ALY monoclonal antibody (CellSignaling THOC4/ALY (D3R4R) Rabbit mAb #12655). Sections were deparaffinized with xylene and hydration with ethanol. For antigen retrieval, the slides were heated with natriumcitrat-puffer (0,1M; pH6,0) for 40 min in a microwave oven. The endogenous peroxidase activity was quenched by a 15 min incubation in a mixture of 3% hydrogen peroxide solution in 100% methanol. Before reaction overnight with the ALY antibody (1:1000)at 4°C in a moist chamber, the sections were blocked with an UltraVision Protein-Block (ThermoScientific: UltraVision LP Large Volume Detektion System HRP Polymer). After incubation with the primary antibody, the slide were washed three times for 5min in PBS. Then the sections were incubated with Primary antibody Enhancer (ThermoScientific: UltraVision LP Large Volume Detektion System HRP Polymer) for 10 min, washed three times for 5min in PBS. For the treatment we get the Large Volume HRP-Polymer (ThermoScientific: UltraVision LP Large Volume Detektion System HRP.Polymer) for 30 min in the dark, washed three times for 5min in PBS. Followed by color development in 3,3´diaminobenzidine tetrahydrochloride (DAKO).Then the slides were counterstained with hematoxylin, dehydrated with ethanol, cleaned with xylol and mounted .

mRNA stability assay

For evaluation of mRNA stability 150.000 SUM159 cells/well were seeded in 12-well plates and transfected with 20 nM siRNA against ALYREF or negative control siRNA as described in section 2.3. After 24 hours, cells for time point 0 (=TP0) were washed with PBS, collected in 750 µl Trizol and stored at -20°C until further use. The remaining wells were treated with actinomycin D (Tocris Bioscience, Abingdon, UK) at a final concentration of 5 µg/ml and further incubated at 37°C, 5% CO_2_. At the indicated time points (i.e. 1, 2, 4, 6, 8 hours) cells were washed with PBS and collected in Trizol, stored at -20°C until all samples were collected. RNA was isolated following a standard Trizol (Invitrogen, Carlsbad, CA, USA) protocol according to the manufacturer’s instructions. mRNA levels were quantified with qRT-PCR as described in section 2.5. Normalize the Ct average of each time point to the Ct average value of t = 0 to obtain ∆Ct value. ∆Ct = (Average Ct of each time point - Average Ct of t=0). Calculate the relative abundance for each time point.

mRNA abundance = 2^(-∆CT)^. Determine the mRNA decay rate by non-linear regression curve fitting (one phase decay) (9).

Cycloheximide chase assay for protein stability

For evaluation of protein stability 450.000 SUM159 cells were seeded in 6-cm dishes and transfected with 20 nM siRNA (either negative scrambled siRNA or ALYREF siRNA) for 48 hours. Afterwards cells were incubated with cycloheximide (Sigma, C7698) at a final concentration of 100 µg/ml. At the indicated time points (0, 1, 2, 5, 8, 24 hours) cells were collected and proteins isolated and subjected to standard Western Blot procedure as described above.

Ethical approval

All animal work was done in accordance with a protocol approved by the Institutional Animal Care and Use Committee at the Austrian Federal Ministry for Science and Research (BMWF) (BMWFW-66.010-0046-WF-V-3b-2016).

Statistical analyses of clinical data

Unpaired or paired Student´s t-test, Fisher’s exact test, Chi-squared test, Spearman correlation, Mann-Whitney and Kruskal-Wallis test were applied where appropriate to analyze the association between ALYREF expression and clinico-pathological parameters. Data of gene expression was log2-transformed. Overall survival was defined as the time from date of diagnosis to the date of death by any cause, and it was assessed using the Kaplan-Meier method. For survival analysis, we separated patients into low/high groups using the median expression value as the cut-off value. Multivariate Cox proportional hazards models including age, primary tumor size, grading, immunohistochemical subtype (estrogen, Her2 and triple negative), Ki67 proliferation index tumor stage (according to the AJCC/UICC 2010 TNM classification) and ALYREF mRNA expression levels. To test the proportional Hazard assumption in cox models Schoenfeld residuals test was used. The reported results included hazard ratios (HR) and 95% confidence intervals (CI). The log-rank test was performed to compare the survival curves of individual groups. A two-sided p<0.05 was considered statistically significant.

References

1. Clemson CM, Hutchinson JN, Sara SA, Ensminger AW, Fox AH, Chess A, et al. An architectural role for a nuclear noncoding RNA: NEAT1 RNA is essential for the structure of paraspeckles. Mol Cell. 2009;33(6):717-26.

2. Livak KJ, Schmittgen TD. Analysis of relative gene expression data using real-time quantitative PCR and the 2(-Delta Delta C(T)) Method. Methods. 2001;25(4):402-8.

3. Lombardo Y, de Giorgio A, Coombes CR, Stebbing J, Castellano L. Mammosphere formation assay from human breast cancer tissues and cell lines. J Vis Exp. 2015(97):doi: 10.3791/52671.

4. Dobin A, Davis CA, Schlesinger F, Drenkow J, Zaleski C, Jha S, et al. STAR: ultrafast universal RNA-seq aligner. Bioinformatics. 2013;29(1):15-21.

5. Liao Y, Smyth GK, Shi W. featureCounts: an efficient general purpose program for assigning sequence reads to genomic features. Bioinformatics. 2014;30(7):923-30.

6. Love MI, Huber W, Anders S. Moderated estimation of fold change and dispersion for RNA-seq data with DESeq2. Genome Biol. 2014;15(12):550.

7. Lellahi SM, Rosenlund IA, Hedberg A, Kiaer LT, Mikkola I, Knutsen E, et al. The long noncoding RNA NEAT1 and nuclear paraspeckles are up-regulated by the transcription factor HSF1 in the heat shock response. J Biol Chem. 2018;293(49):18965-76.

8. Murthy UM, Rangarajan PN. Identification of protein interaction regions of VINC/NEAT1/Men epsilon RNA. FEBS Lett. 2010;584(8):1531-5.

9. Ratnadiwakara M, Änkö M. mRNA Stability Assay Using Transcription Inhibition by Actinomycin D in Mouse Pluripotent Stem Cells. Bio-Protocol. 2018;8(21):e3072.
