# Supplementary material for: ALYREF, a novel factor involved in breast carcinogenesis, acts through transcriptional and post-transcriptional mechanisms selectively regulating the short NEAT1 isoform
Source: Cell Mol Life Sci. 2022 Jul 1;79(7):391. doi: 10.1007/s00018-022-04402-2 (PMC9249705; doi:10.1007/s00018-022-04402-2)
Supplement: Supplementary file 2 — Supplementary file2 (DOCX 21 KB) [file 18_2022_4402_MOESM2_ESM.docx]

**SUPPLEMENTARY FIGURE LEGENS**

**Figure S1 | Relevance of ALYREF genomic alterations and expression levels in human breast cancer.**

**(A)** Alteration frequency of ALYREF in breast cancer patients of four different cohorts (n=3989) indicates average amplification frequency of 5%.

**(B)** RNAseq data showing the expression of ALYREF in TNBC (n=317) vs. non-TNBC (n=4119) cancer tissue samples. Data were derived from the publicly available bcgenex miner database.

**(C)** Representative pictures of ALYREF protein expression in tissue of breast cancer patients. Data have been derived from the publicly available database ([https://www.proteinatlas.org/ENSG00000183684-ALYREF/pathology/breast+cancer#img](https://www.proteinatlas.org/ENSG00000183684-ALYREF/pathology/breast+cancer)).

**(D)** ALYREF protein expression among different breast cancer subtypes created by using the publicly available database http://www.breastcancerlandscape.org/.

**(E)** Western Blot comparing ALYREF expression between several TNBC cell lines and normal mammary epithelial cells. B-Actin was used as a loading control and results were quantified densitometrically in relation to the normal mammary cell line.

**(F)** Results of staining intensity of ALYREF expression in invasive breast carcinoma samples (n=100) and adjacent normal breast tissue samples (n=10) on a commercially available tissue microarray. Graph depicts IHC intensity scores from 0, 1, 2 to 3, mean±SD, ***p<0.001.

**(G)** Kaplan-Meier curve showing overall survival of breast cancer patients with high or low ALYREF protein expression (Tang_2018 dataset derived from Kaplan Meier plotter). HR=2.99 (CI 1.32-6.76), p=0.0059.

**Figure S2 | Quantification of siRNA-mediated knock-down efficiency of ALYREF on mRNA and protein level upon 72 to 96 hours.**

**(A-C)** Quantification of knock-down efficiency of ALYREF in different breast cancer cell lines after treatment with specific siRNAs against ALYREF or negative control siRNA 72 h after transfection on mRNA level via real-time PCR using GAPDH as reference gene. n=3, ±SD. *p<0.05, **p<0.01, ***p<0.001.

**(D-F)** Quantification of ALYREF knock-down efficiency on protein level via Western Blot using β-Actin as loading control 72 or 96 hours after transfection.

**Figure S3** **| Quantification of siRNA-mediated knock-down efficiency of ALYREF on mRNA and protein level upon 24 to 48 hours**

**(A-C)** Quantification of knock-down efficiency of ALYREF in different breast cancer cell lines after treatment with specific siRNAs against ALYREF or negative control siRNA 24 and 48 h after transfection on mRNA level via real-time PCR using GAPDH as reference gene. n=3, ±SD. *p<0.05, **p<0.01, ***p<0.001.

**(D-F)** Quantification of ALYREF knock-down efficiency on protein level via Western Blot using β-Actin as loading control 24 or 48 hours after transfection.

**Figure S4 | *In vitro* phenotypic characterization of ALYREF silencing in three other cell lines.**

**(A-C)** WST-1 cell growth assay in TNBC cell lines over 96 hours under control conditions (Negative Control siRNA; grey curve) or after siRNA-mediated knock-down of ALYREF (blue curves). n=6, ±SD. ***p<0.001.

**(D-F)** Colony formation assay in three TNBC cell lines. Bar graphs on the left represent relative colony numbers under control conditions (Negative Control siRNA; white bars) or after siRNA-mediated ALYREF knock-down (blue bars). n=3, ±SD. **p<0.01, ***p<0.001. *Right panels* depict corresponding representative pictures.

**(G-I)** Mammosphere formation was analyzed either under control conditions (Negative Control siRNA; white bars) or after siRNA-mediated knock-down of ALYREF (blue bars) ten days after transfection. The relative numbers of spheres in ALYREF silenced compared to control conditions are represented in the graphs. n=3, ±SD. ***p<0.001.

**Figure S5 |** **ALYREF knock-down reduces anchorage independent growth in TNBC cells.**

**(A,B)** Soft agar assay to determine the anchorage independent growth of SUM159 cells. Graphs depict the relative numbers of colonies counted either under control conditions (Negative Control siRNA, white bars) or after siRNA-mediated knock-down of ALYREF (blue bars). n=3, ±SD. ***p<0.001.

**Figure S6** **| Phenotypic characterization of stably ALYREF overexpressing SUM159 cells vs control SUM159 cells.**

**(A,B)** Evaluation of ALYREF expression levels on (A) mRNA level via qRT-PCR using specific primers for ALYREF. GAPDH was used as reference gene. n=3, ±SD, (B) or on protein level with Western Blot whereas β-actin was used as loading control. n=4. Lanes 1-4: ALYREF overexpression, lanes 5-8: control.

**Figure S7** **| ALYREF silencing induces apoptosis in TNBC cells.**

**(A-C)** Caspase 3/7 assay either under control conditions (Negative Control siRNA, white bars) or after siRNA-mediated knock-down of ALYREF (blue bars) 72 and 96 hours after transfection. n=3, ±SD. ***p<0.001.

**(D-F)** Western Blot analysis of PARP shows the ratio of cleaved PARP to full-length PARP in ALYREF silenced cells in the four TNBC cell lines 72 hours after transfection. β-Actin was used as loading control and over-night staurosporine (1 µM) treatment as positive control.

**(G)** Flow-cytometry based AnnexinV staining of SUM159 cells either under control conditions or 48 hours after siRNA-mediated silencing of ALYREF. Transfection with Allstar cell death control siRNA has been used as positive apoptosis control.

**(H)** Bar chart diagram summarizing the number of Annexin positive cells upon in siRNA control, Alyref siRNA and Allstar cell death control. n=3. ***p<0.001.

**Figure S8 | ALYREF knock-down does not influence mitochondria count or volume.**

(**A,B**) Corresponding statistical analysis to Fig. 3E of mitochondrial count (**A**) and mitochondrial volume (**B**). n=6, ±SD.

**Figure S9** **| Phenotypic *in vitro* characterization of inducible ALYREF silencing.**

**(A)** Three different ALREF inducible-shRNA clones (labeled as clone #1, #2 and #3) were tested and protein levels of ALYREF were detected upon 72 hours of doxycycline-induced shRNA knock-down. ALYREF protein expression was significantly decreased in clone #1 and #3, which were chosen for further downstream assays (and renamed as clone A and clone B in the following experiments).

**(B)** Alyref mRNA expression was measured in those two clones (A and B) by qRT-PCR using U6 as a housekeeper in comparison to the control shRNA clone. n=3, ±SD

**(C,F)** WST-1 cellular growth assay of SUM159 clones A and B carrying inducible shRNA ALYREF lentiviral constructs compared to control cells under conditions (C) without doxycycline induction or (F) with doxycycline induction. n=6, ±SD.

**(D,G)** Bars show results of CFU assay in stably ALYREF silenced SUM159 cells compared to control cells (D) without doxycycline induction or (G) with doxycycline induction.

**(E,H)** Representative images of CFU assay either (E) without doxycycline induction or (H) with doxycycline induction. n=3, ±SD. ***p<0.001.

**Figure S10 | ALYREF silenced cancer cells inhibit TNBC growth *in vivo*.**

**(A-C)** Stably transfected inducible GFP-shRNA ALYREF-silenced SUM159 cells (clone B; left side) or control shRNA SUM159 (clone B; right side) were subcutaneously injected at a density of 1x10^6^ cells into the mammary fat pad of the mice. For induction of ALYREF silencing doxycycline was administered through the drinking water (200 µg/ml). n=6. (**A**) Optical in vivo imaging of the whole body measuring the GFP intensity of xenograft tumors. (**B**) On day 55 mice were sacrificed and tumors were dissected. Corresponding optical imaging of the tumors. Upper panel shows dissected tumors and lower panel optical imaging of GFP signals. (**C**) Corresponding statistical analysis of tumor volumes. n=6, ±SD. *p<0.05. **(D)** Representative image of immunohistochemical analysis of the xenograft tumors in control mice showing a strong rather nuclear expression of ALYREF protein expression.

**Figure S11 | NEAT1_2 does not influence growth or apoptosis of TNBC cells.**

**(A,B)** Quantification of NEAT1_1 and ALYREF expression levels in stable ALYREF overexpressing cell lines via qRT-PCR using GAPDH and U6 as housekeeping genes. n=3, ±SD. *p<0.05.

(**C**) Knock-down efficiency of NEAT1 in the indicated cell lines after transfection with siRNA against NEAT1_1 or NEAT1_2 or control siRNA via qRT-PCR. GAPDH was used as reference gene. n=3, ±SD.

(**D,E**) Cellular growth assay in TNBC cell lines over 96 hours under control conditions (Negative Control siRNA) or after siRNA-mediated knock-down of NEAT1_2. n=6, ±SD.

(**F,G**) Caspase 3/7 assay either under control conditions (Negative Control siRNA) or after siRNA-mediated knock-down of NEAT1_2 96 hours after transfection. n=3, ±SD. **p<0.01, ***p<0.001.

**Figure S12 | NEAT1_1 knock-down alters mitochondrial metabolism and ATP production.**

**(A,B)** *(Left panels)* OCR under control conditions (black curves) or after siRNA-mediated silencing of NEAT1_1 (red curve) as well as under control conditions (black curves). OCR was normalized to protein content. As indicated, cells were treated with 2 µM oligomycin, 0.2 µM FCCP and 5 µM antimycin. (*Right panels*) corresponding statistical analysis of basal and maximal respiration.

(**C,D**) Corresponding statistical analysis of mitochondrial ATP production. n=3. Bars represent mean ± SEM. *p<0.05, **p<0.01.

**Figure S13 | NEAT1_1 knock-down influences mitochondrial morphology.**

**(A)** Representative pictures of mitochondrial morphology of SUM159 cells stained with mitoTracker® red FM for 30 minutes under control conditions (negative control siRNA) or after siRNA-mediated silencing of NEAT1_1.

**(B)** Corresponding statistical analysis of mitochondrial elongation. n=6, ±SD. *p<0.05.

**Figure S14 | NEAT1_1 knock-down does not influence mitochondrial count or volume.**

(**A,B**) Corresponding statistical analysis to Fig. S13 of mitochondrial count (**A**) and mitochondrial volume (**B**). n=6, ±SD. Same control as in Fig. 3E,F has been used.

**Figure S15 NEAT1 RNA in-situ hybridization (FISH) upon ALYREF and NEAT1 knock-down and ALYREF overexpression (A,B)** Representative images of fluorescence in-situ hybridization (FISH) detecting NEAT1 (red signal) with an RNA probe specific against NEAT1 (pan-variant) in SUM159 (A, upper panel) or MDA-MB-231 (B, lower panel) cells either under control conditions (left panels), after knock-down of ALYREF (middle panel) or after silencing of NEAT1 (right panel) with specific siRNAs. Dapi was used for visualizing the nuclei. **(C,D)** Corresponding statistical analysis of mean amount of NEAT signals per cell in SUM159 (upper panel) or MDA-MB-231 (lower panel) cells under the same conditions. As shown, both, the ALYREF knock-down and as a control, the NEAT1 knock-down led to significant decrease of NEAT1 signals per cell. **(E)** Representative images of FISH detecting NEAT1 (red signal) with an RNA probe specific against NEAT1 in SUM159 Control cells (left panel) or SUM159 ALYREF stable overexpressing cells (right panel). Dapi was used for visualizing nuclei. **(F)** Corresponding statistical analysis of mean amount of paraspeckles per cell. Bars represent mean ± SD (n>5), **p<0.01.n>5, ±SD. *p<0.05.

**Figure S16 | ALYREF binds NEAT1 RNA.**

(**A**) Purified RNA was analyzed with standard PCR with NEAT1 specific primers after RIP procedure detecting specific bands in the input, positive control (p54nrb/NONO) and the ALYREF sample. No bands were detected in the negative control and no-template control samples.

(**B**) Western blot analysis of lysates detecting ALYREF before RIP procedure (left panel – Input sample) and after RIP procedure (middle panel) and detecting p54nrb/NONO as positive control (right panel).

(**C**) Statistical analysis of RIP using ALYREF antibody detecting no interaction with NEAT1_2.

**Figure S17 | ALYREF does not influence stability of NEAT1_2.**

mRNA stability assay in SUM159 cells treated with actinomycin D either under control conditions (Negative control siRNA) or after ALYREF silencing. (*Left panel*) Graph representing NEAT1_2 decay over a period of 8 hours. (*Right panel*) Corresponding calculated half lives of NEAT1.

**Figure S18**

Relative gene expression of NUDT21 and CPSF6 either under control conditions or after si-RNA mediated silencing of ALYREF. (n=3, *p<0.05, **p<0.01).

**Figure S19 | ALYREF does not influence mRNA stability of CPSF6 or NUDT21.**

mRNA stability assay in SUM159 cells treated with actinomycin D either under control conditions (Negative control siRNA) or after ALYREF silencing.

**Figure S20 | ALYREF stabilizes CPSF6 upon 16 and 20 hours of cycloheximide exposure**

Cycloheximide chase assay and densitometry to assess protein stability of CPSF6 under control conditions or after ALYREF silencing at 16 and 20 hours. Left panel shows a decrease in the CPSF6 protein levels after 16 and 20 hours of cycloheximide exposure, whereas (right panel) in the control condition no decrease could be detected. Cofilin was used as housekeeper.

**Figure S21 | ALYREF does not influence protein stability of NUDT21.**

Cycloheximide chase assay to assess protein stability of NUDT21 under control conditions or after ALYREF silencing over 24 h. Cofilin was used as housekeeper.

**Figure S22 | CPSF6 knockdown efficiency**

**(A,B)** siRNA-mediated silencing efficiency of CPSF6 on mRNA (A) and protein level (B).

**Figure S23 | NUDT21 silencing does not influence NEAT1 isoforms expression**

Evaluation of RNA expression of NUDT21, NEAT1_1 and NEAT1_2 after siRNA-mediated NUDT21 silencing in two TNBC cell lines (n=3, *p<0.05, **p<0.01).

**Figure S24 | Correlation analysis between ALYREF and CPSF6.**

**(A)** Data showing CPSF6 expression in matched tissue samples of breast cancer patients (n=112). RNAseq data derived from the publicly available TNM plotter.

(**B**) Correlation analysis of CPSF6 and ALYREF expression in breast cancer cell lines from all subtypes. Data were derived from the publicly available depmap.org database (n=61, Spearman correlation = 0.634, p<0.0001).

**(C)** Correlation analysis of CPSF6 and ALYREF expression in breast cancer patients. Data were derived from the publicly available bc-genexminer database (n=4712, Pearson correlation = 0.18, p<0.0001).

**(D-G)** Correlation analysis of CPSF6 and ALYREF expression in breast cancer cell lines from different subtypes i.e. Luminal B (D), normal breast-like (E), Her2-enriched (F) and Luminal A (G). Data were derived from the publicly available depmap.org database.
