# Supplementary material for: ALYREF, a novel factor involved in breast carcinogenesis, acts through transcriptional and post-transcriptional mechanisms selectively regulating the short NEAT1 isoform
Source: Cell Mol Life Sci. 2022 Jul 1;79(7):391. doi: 10.1007/s00018-022-04402-2 (PMC9249705; doi:10.1007/s00018-022-04402-2)
Supplement: Supplementary file 3 — Supplementary file3 (PPTX 23516 KB) [file 18_2022_4402_MOESM3_ESM.pptx]

## Slide 1
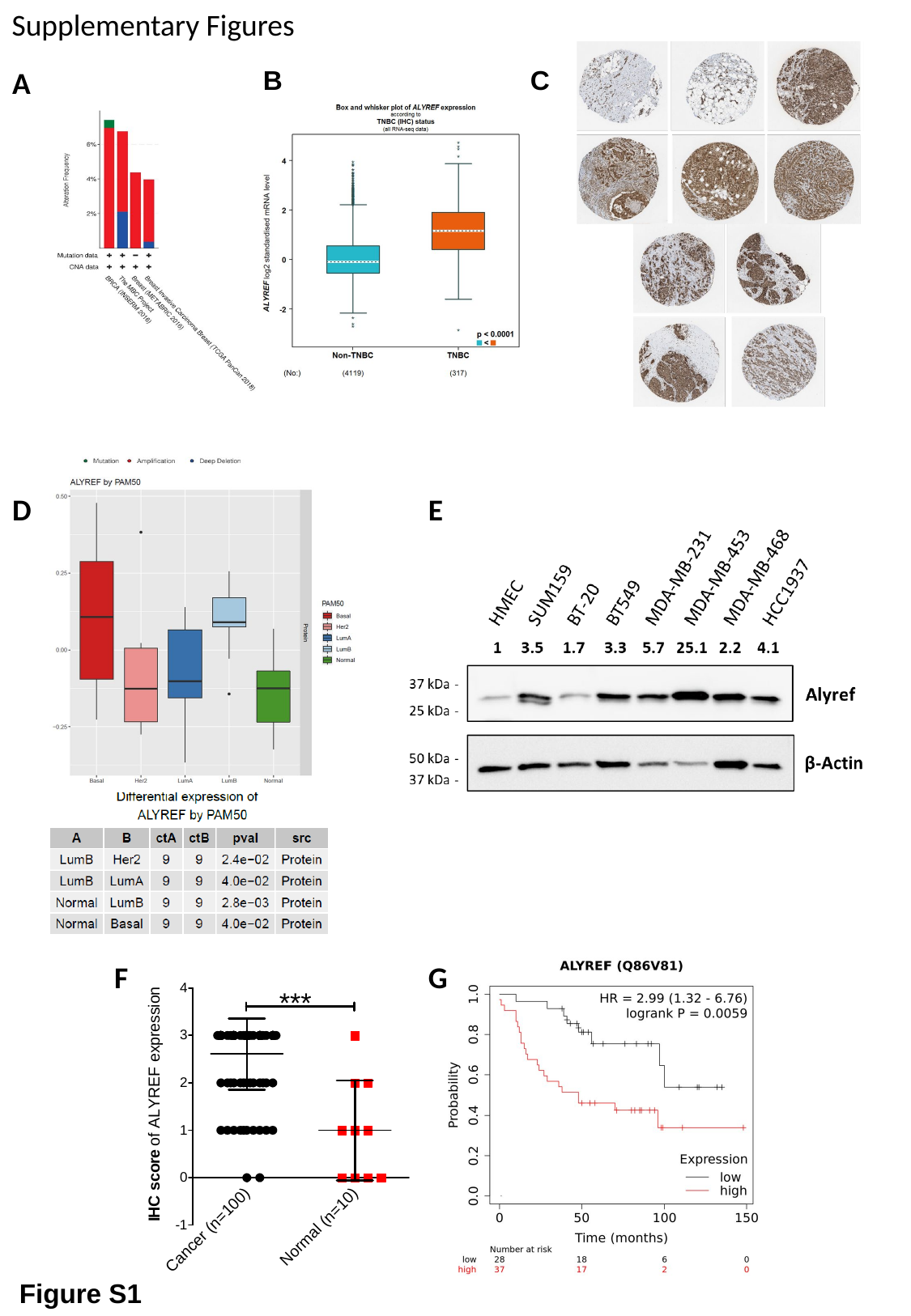

Supplementary Figures
A
B
C
D
E
F
G
 Figure S1

## Slide 2
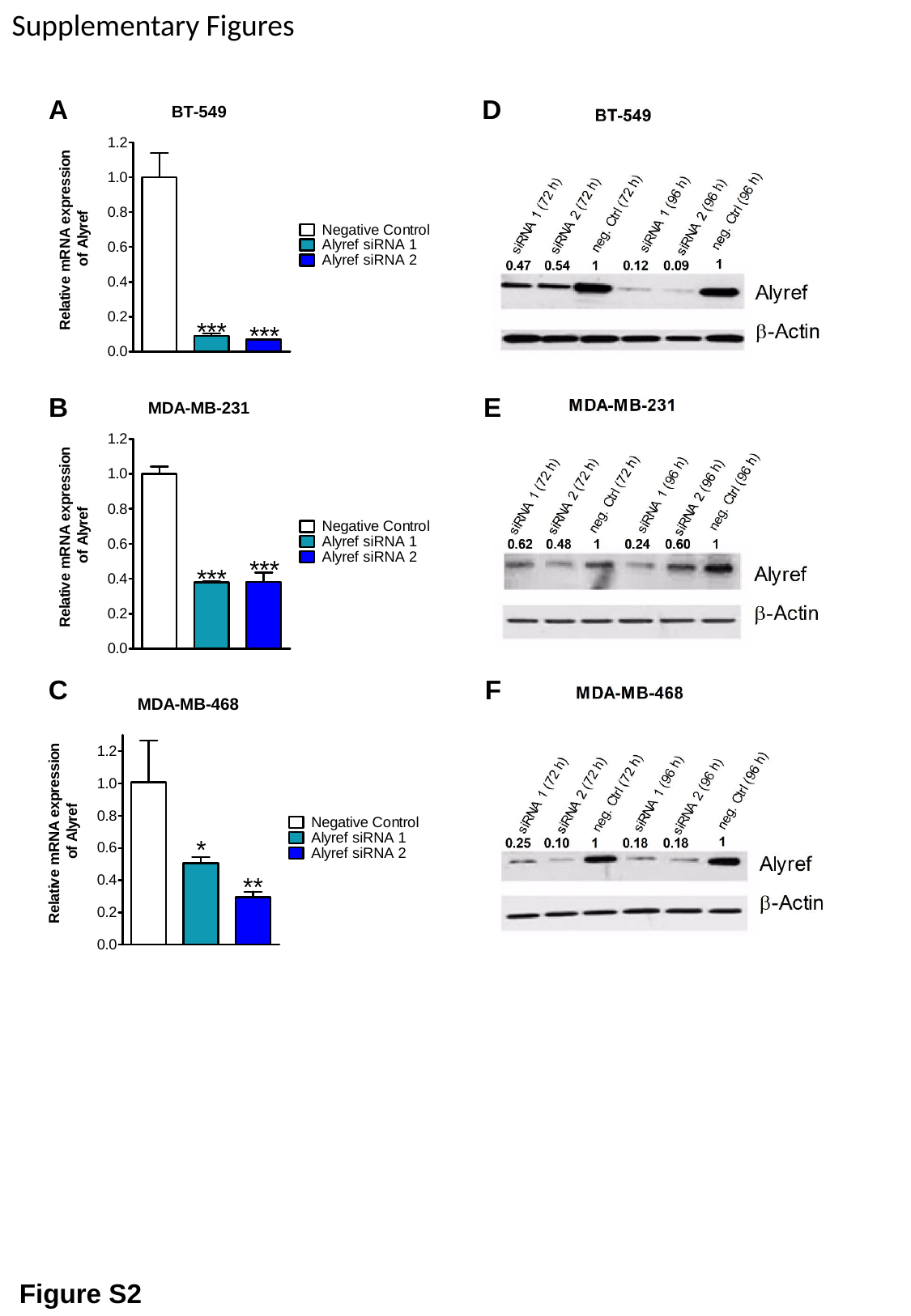

Supplementary Figures
A
D
B
E
C
F
 Figure S2

## Slide 3
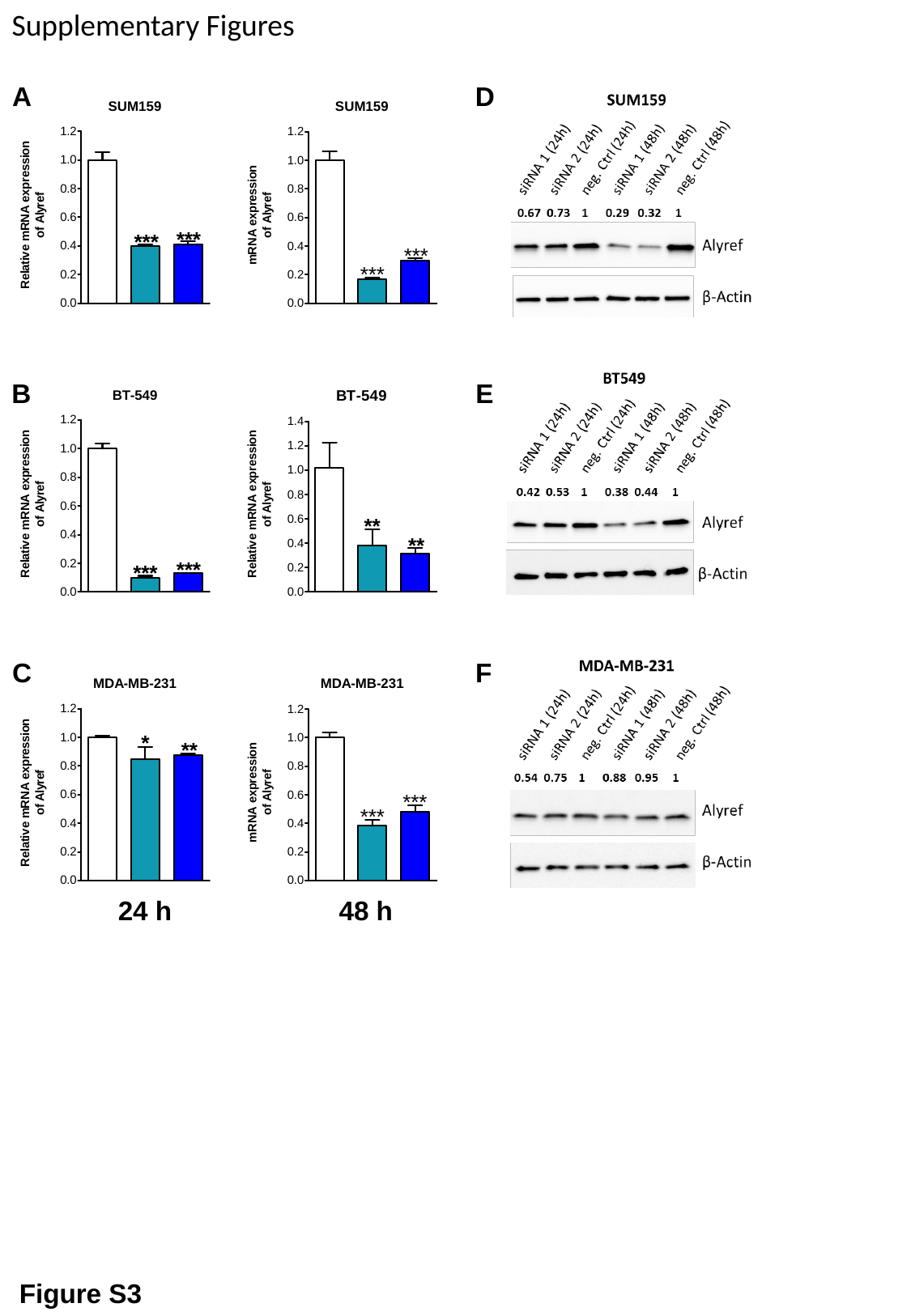

Supplementary Figures
A
D
B
E
C
F
24 h
48 h
 Figure S3

## Slide 4
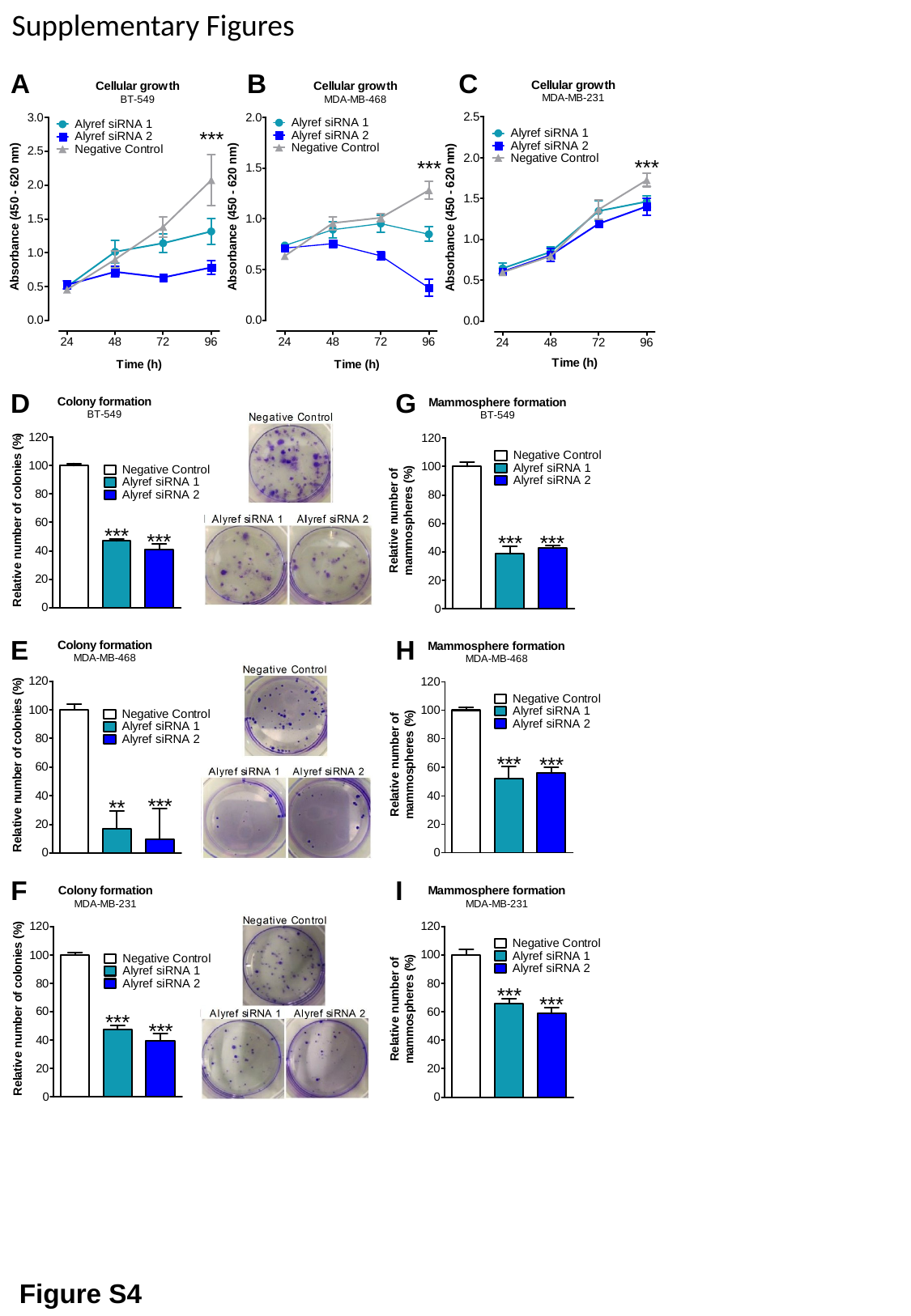

Supplementary Figures
A
B
C
D
G
E
H
F
I
 Figure S4

## Slide 5
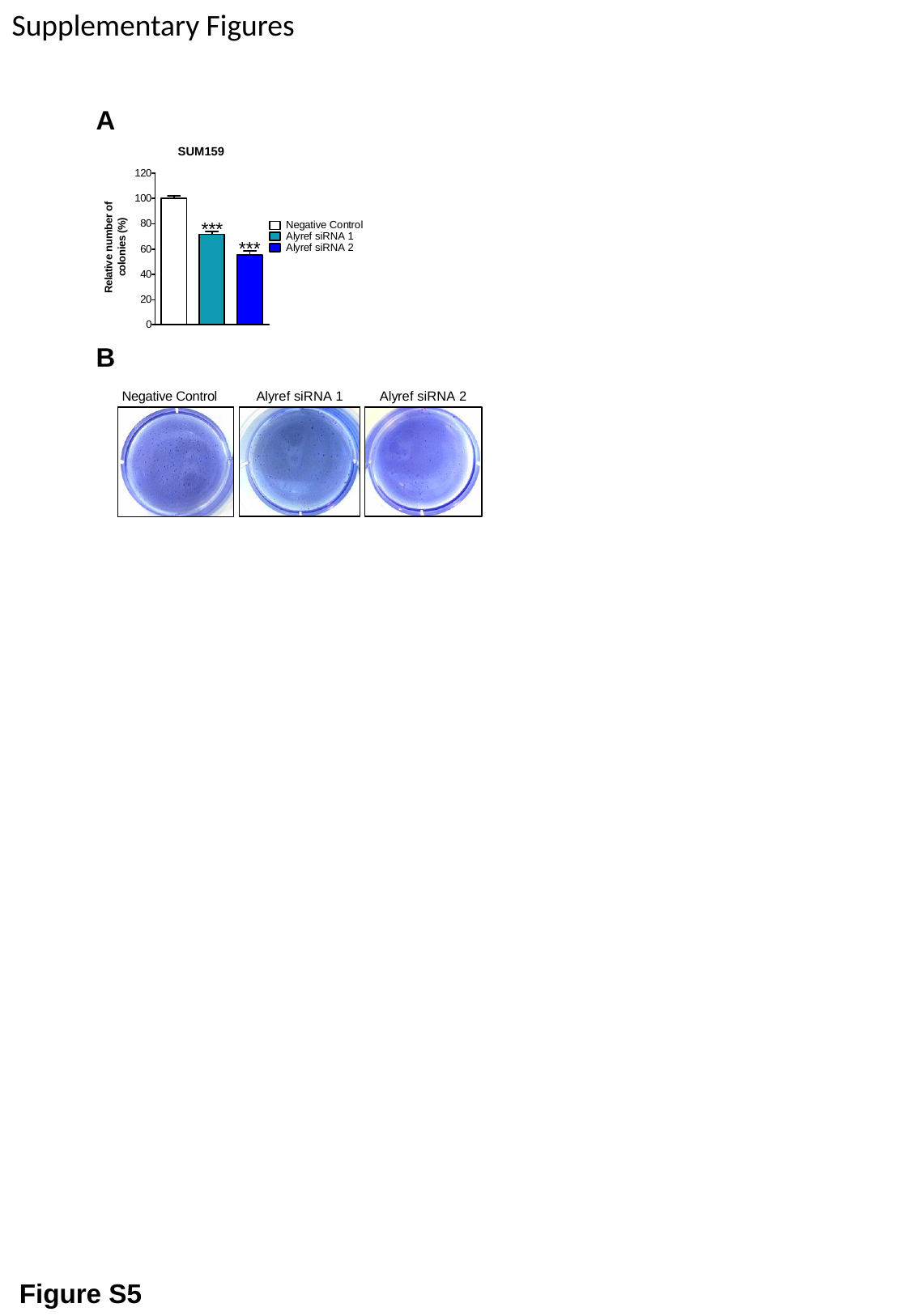

Supplementary Figures
 Figure S5

## Slide 6
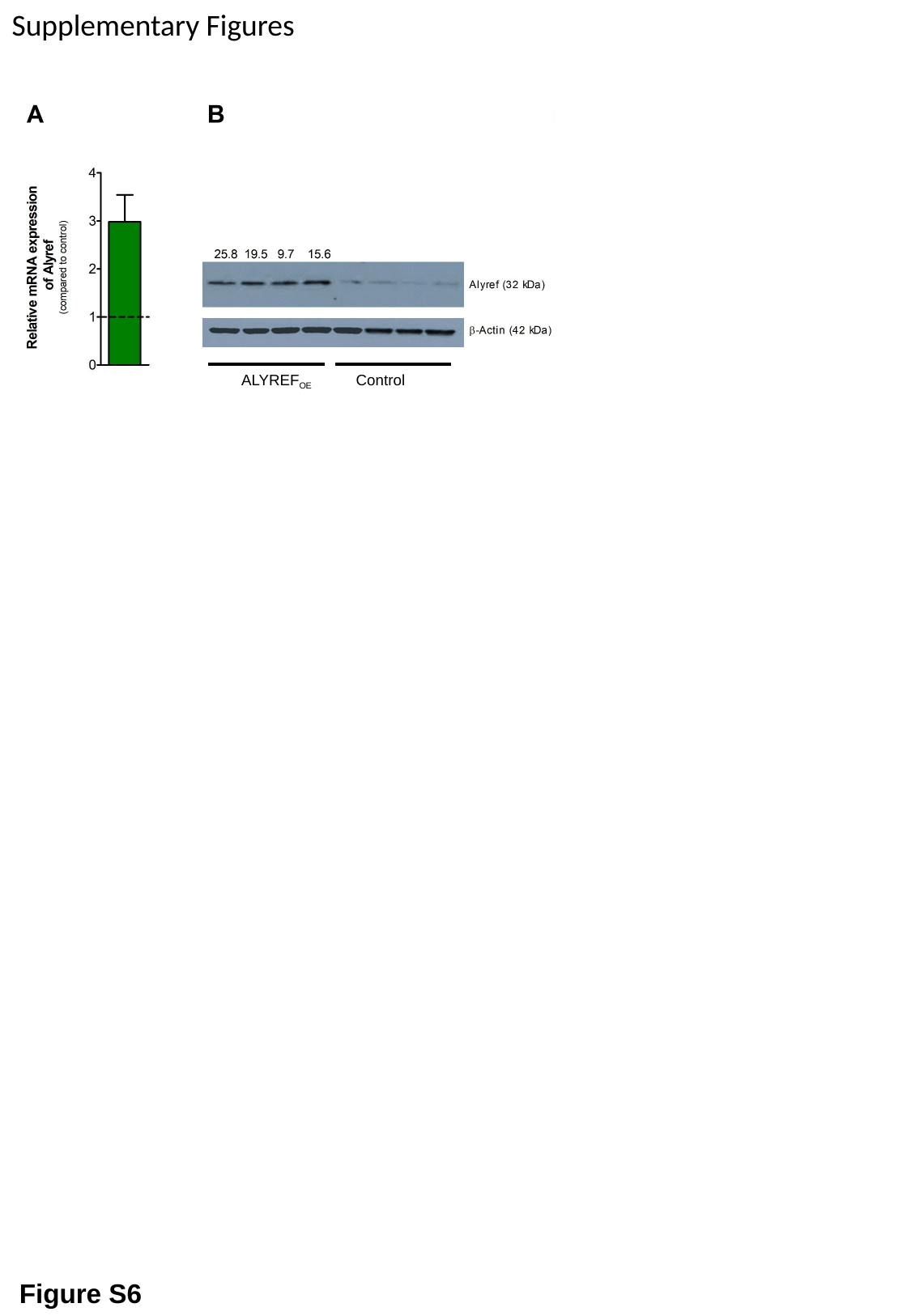

Supplementary Figures
ALYREFOE
Control
 Figure S6

## Slide 7
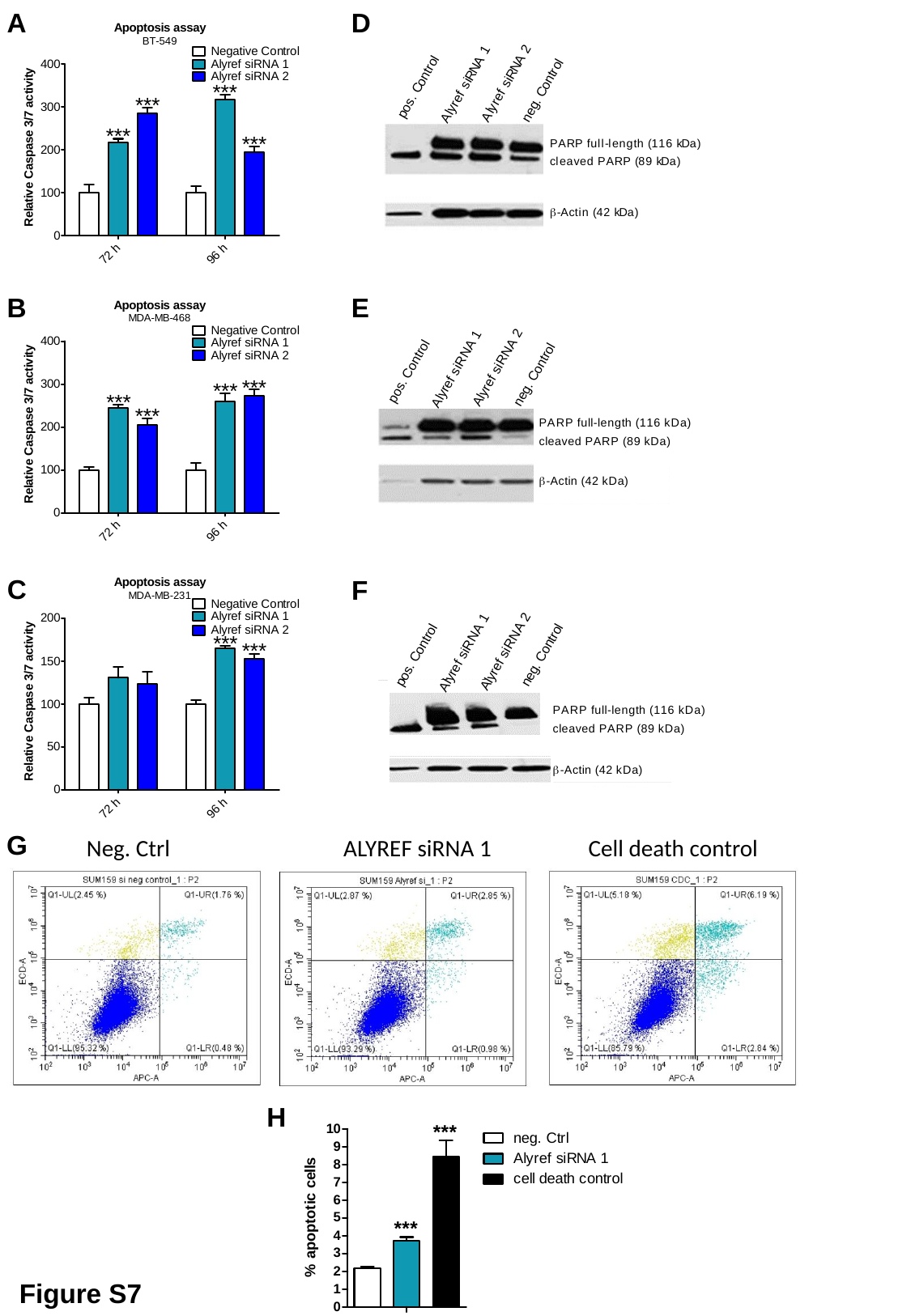

A
D
B
E
C
F
G
Neg. Ctrl
ALYREF siRNA 1
Cell death control
H
 Figure S7

## Slide 8
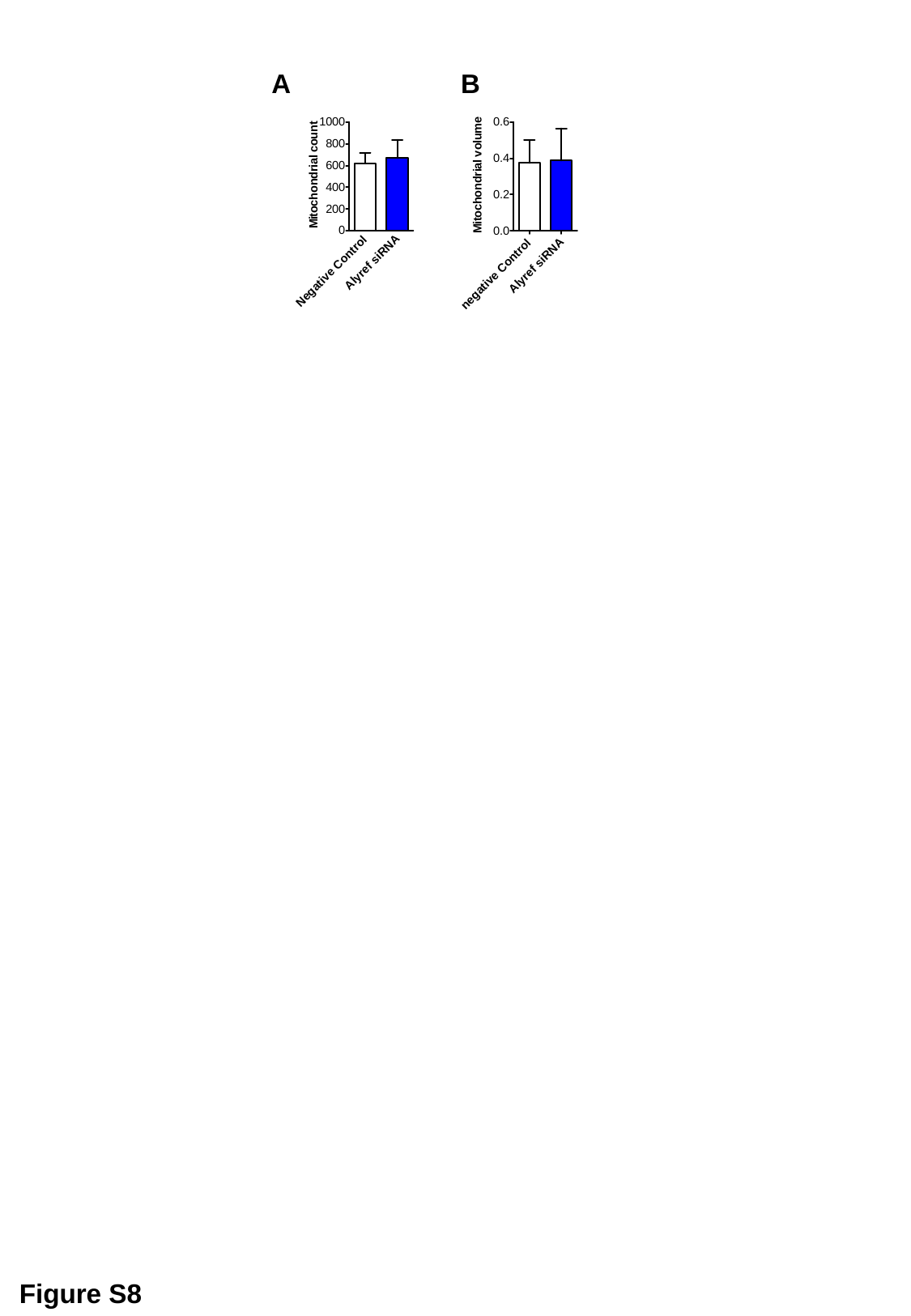

Figure S8

## Slide 9
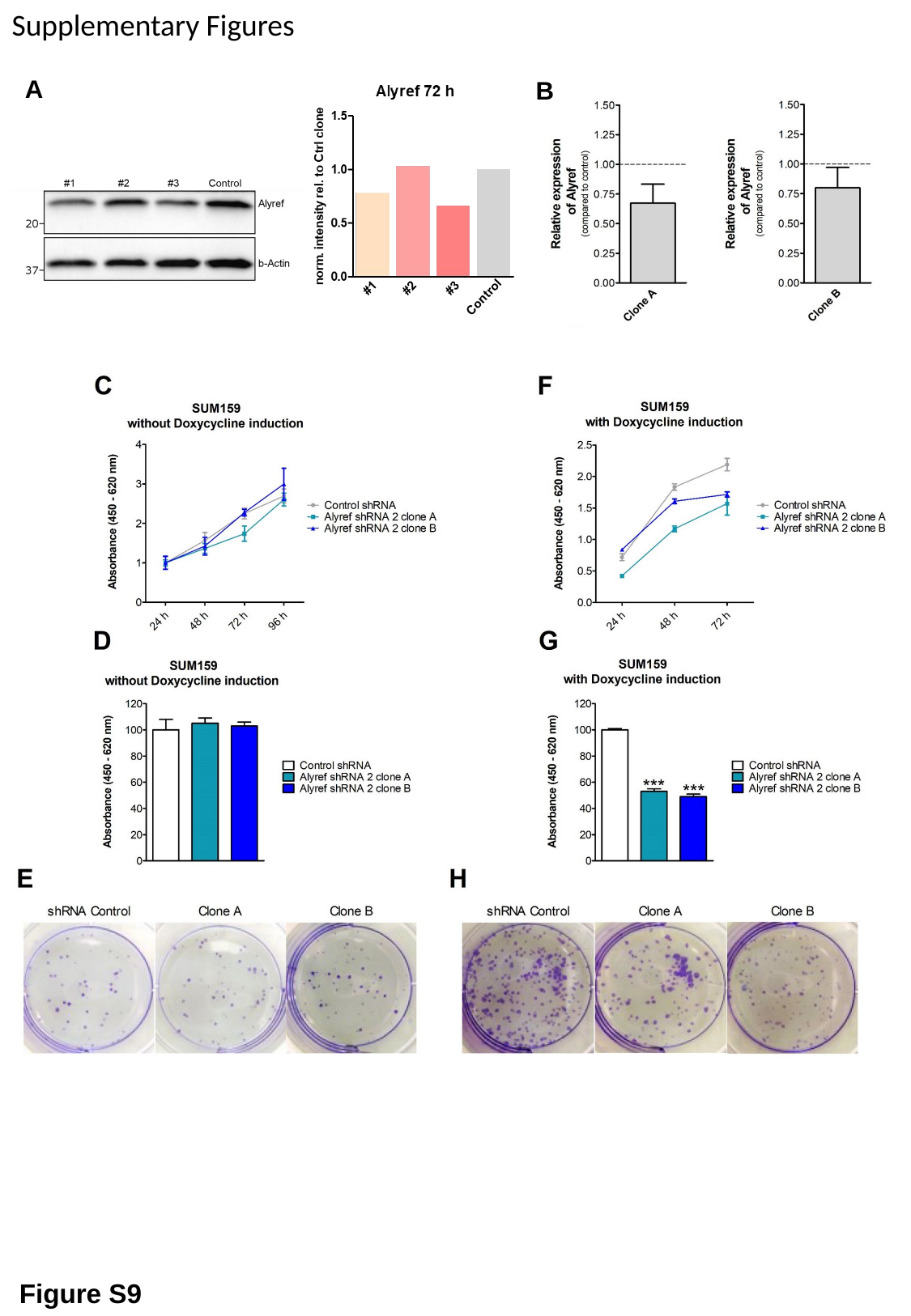

Supplementary Figures
A
B
 Figure S9

## Slide 10
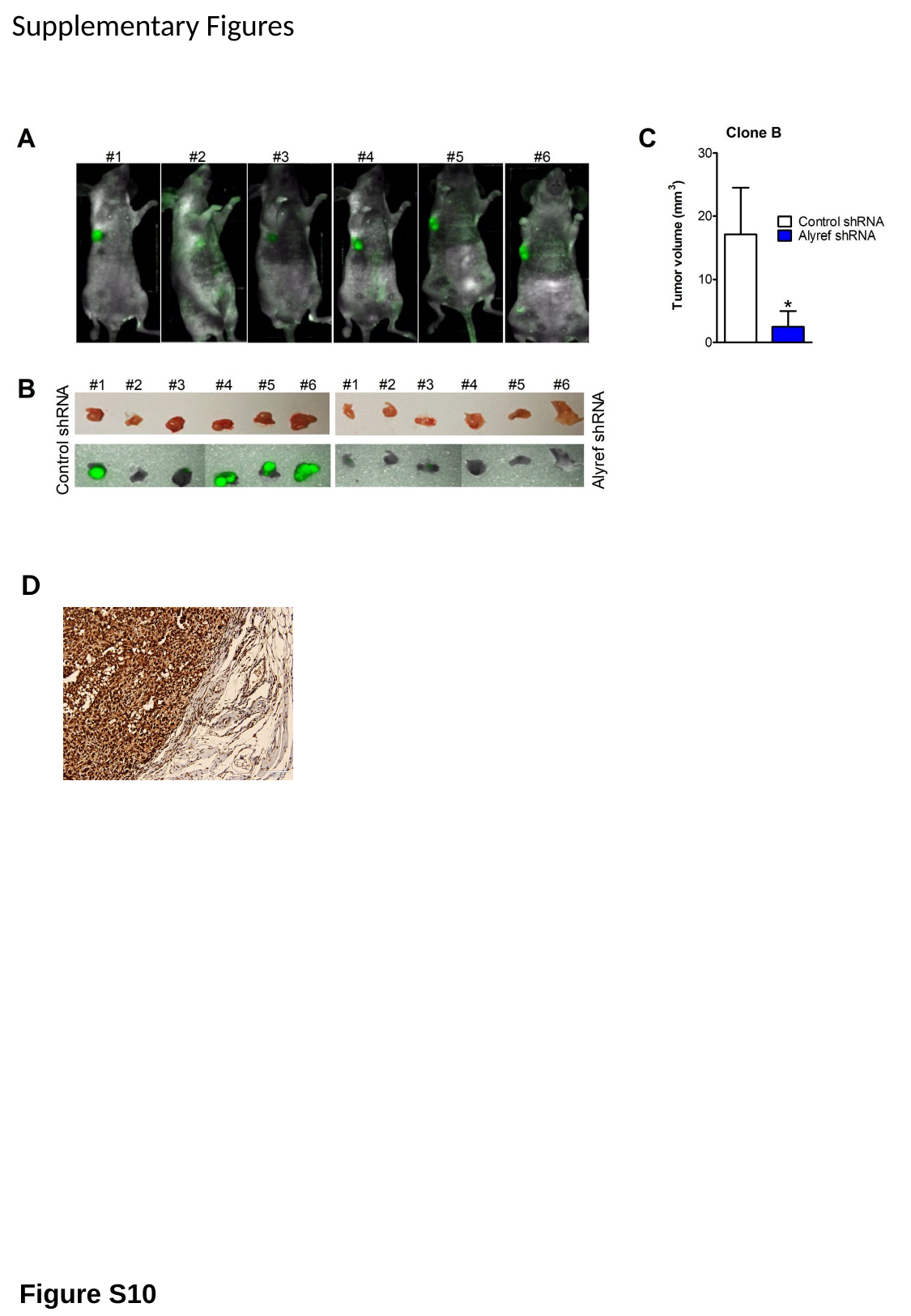

Supplementary Figures
D
 Figure S10

## Slide 11
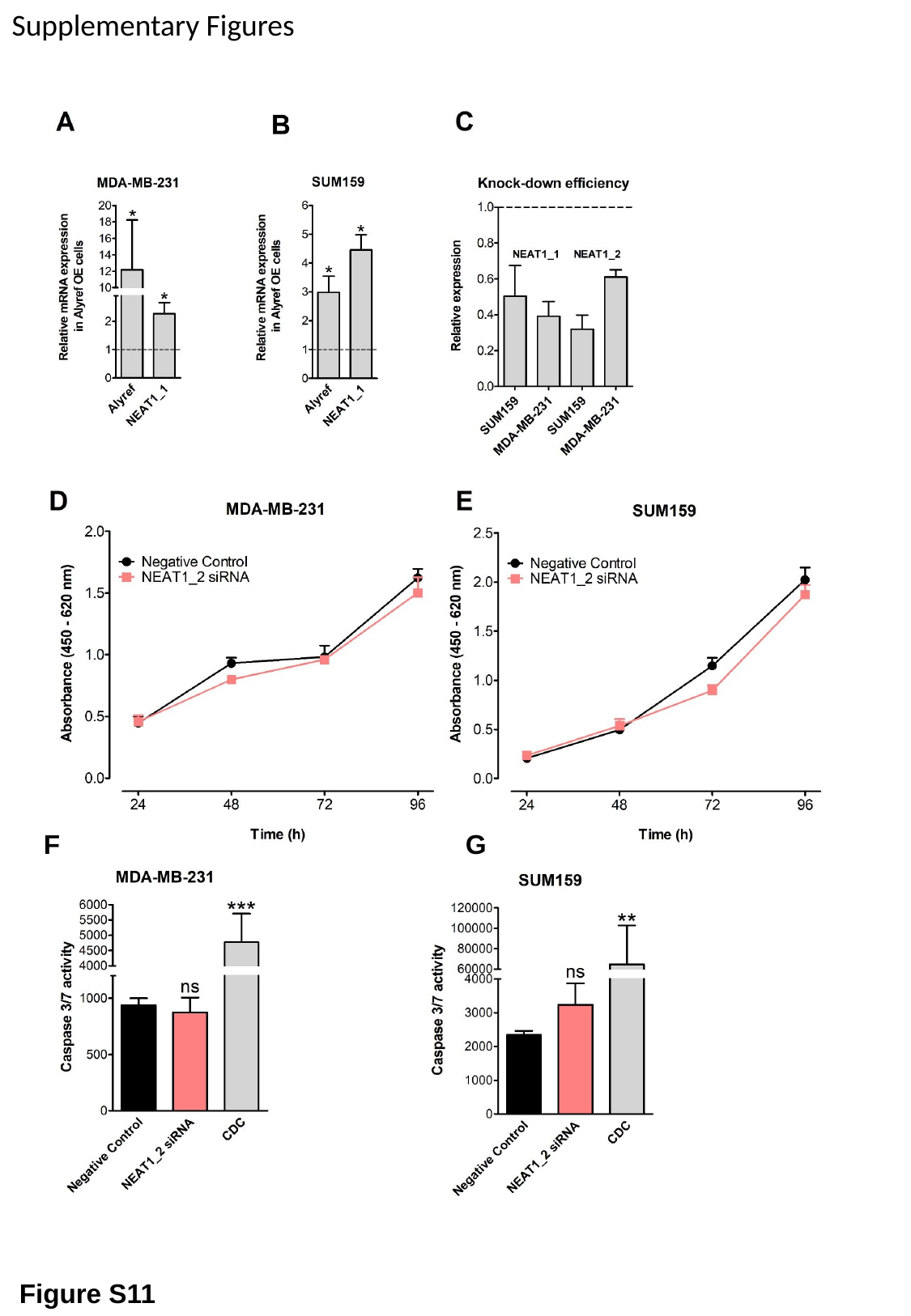

Supplementary Figures
F
G
 Figure S11

## Slide 12
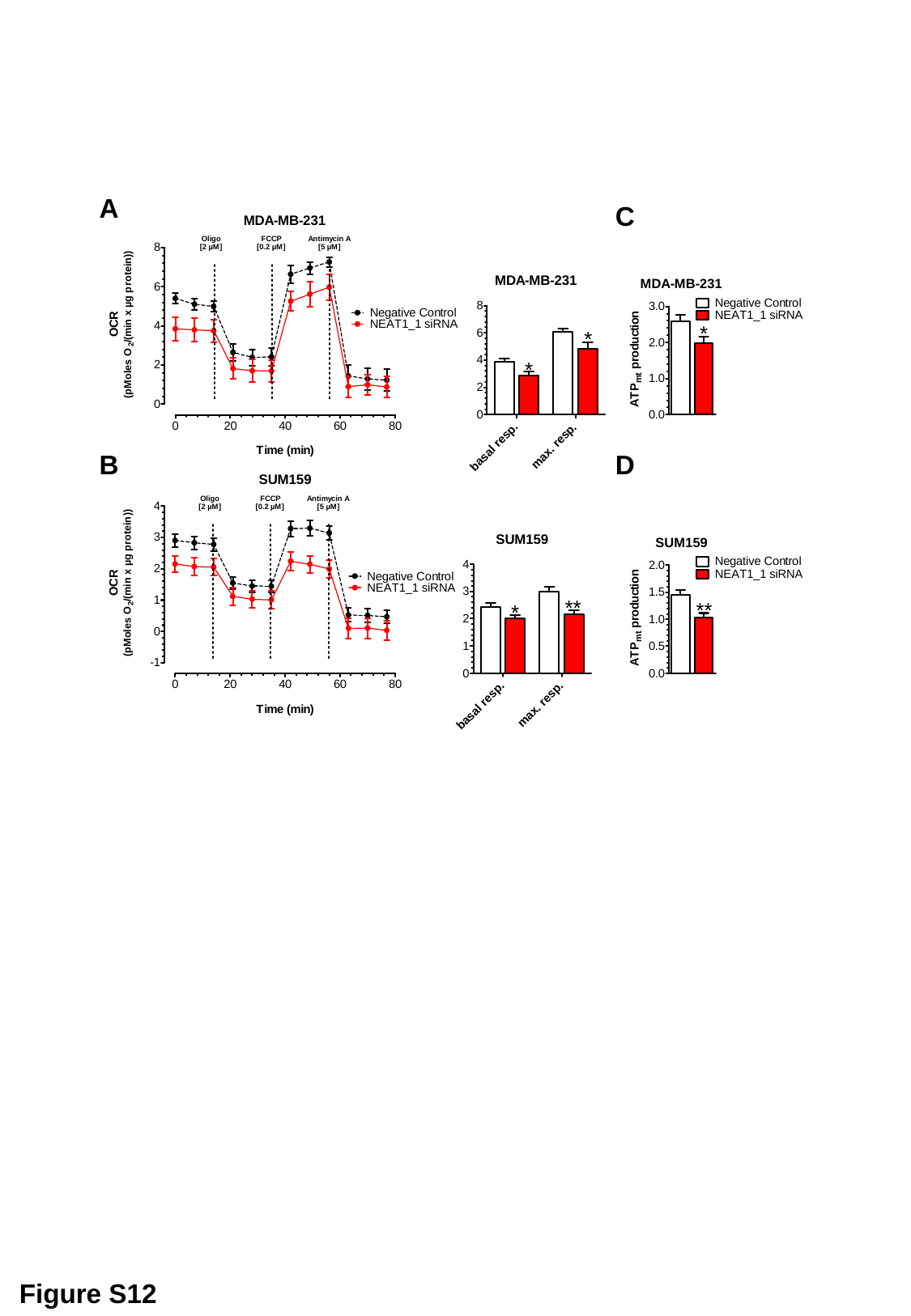

Figure S12

## Slide 13
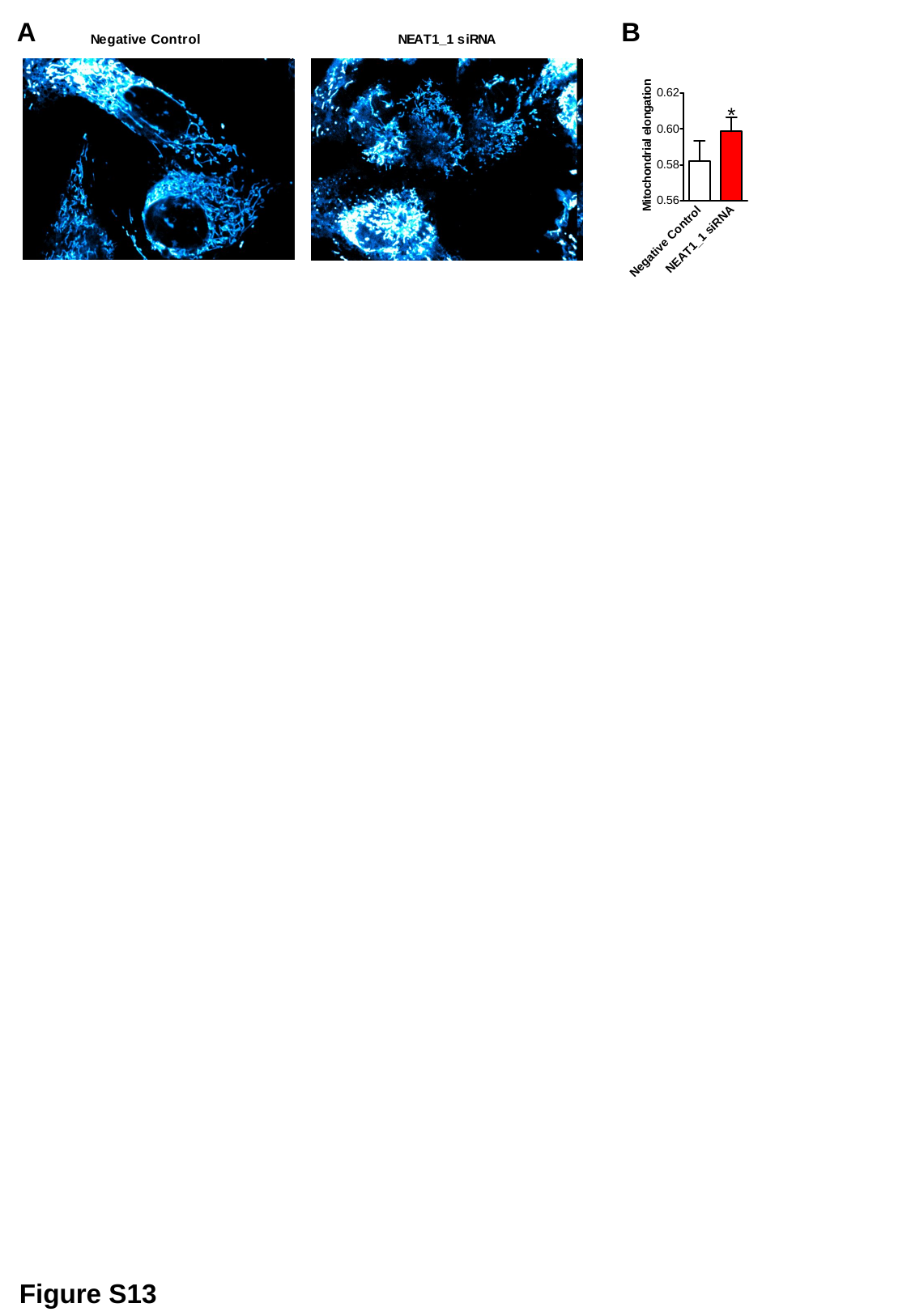

Figure S13

## Slide 14
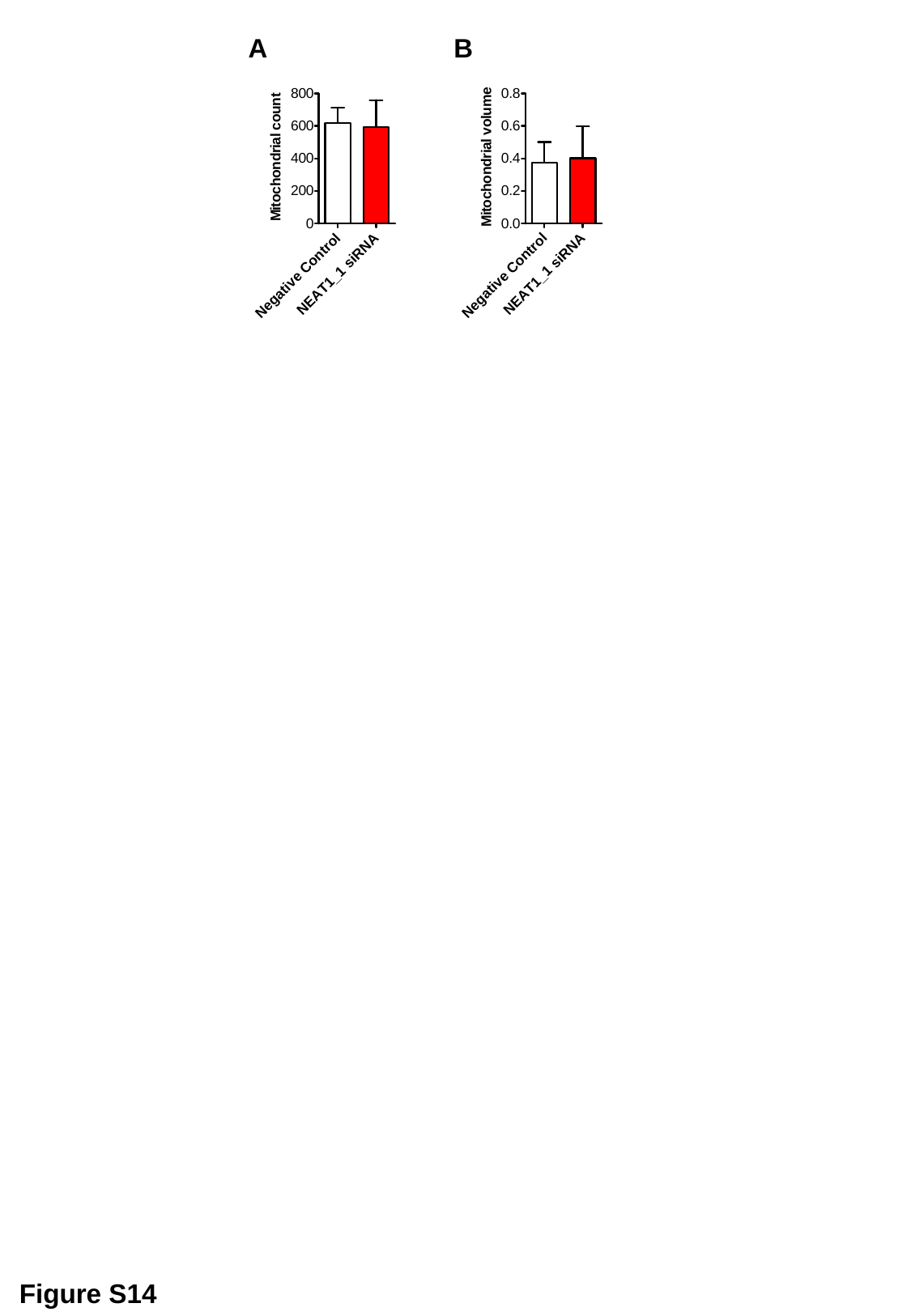

Figure S14

## Slide 15
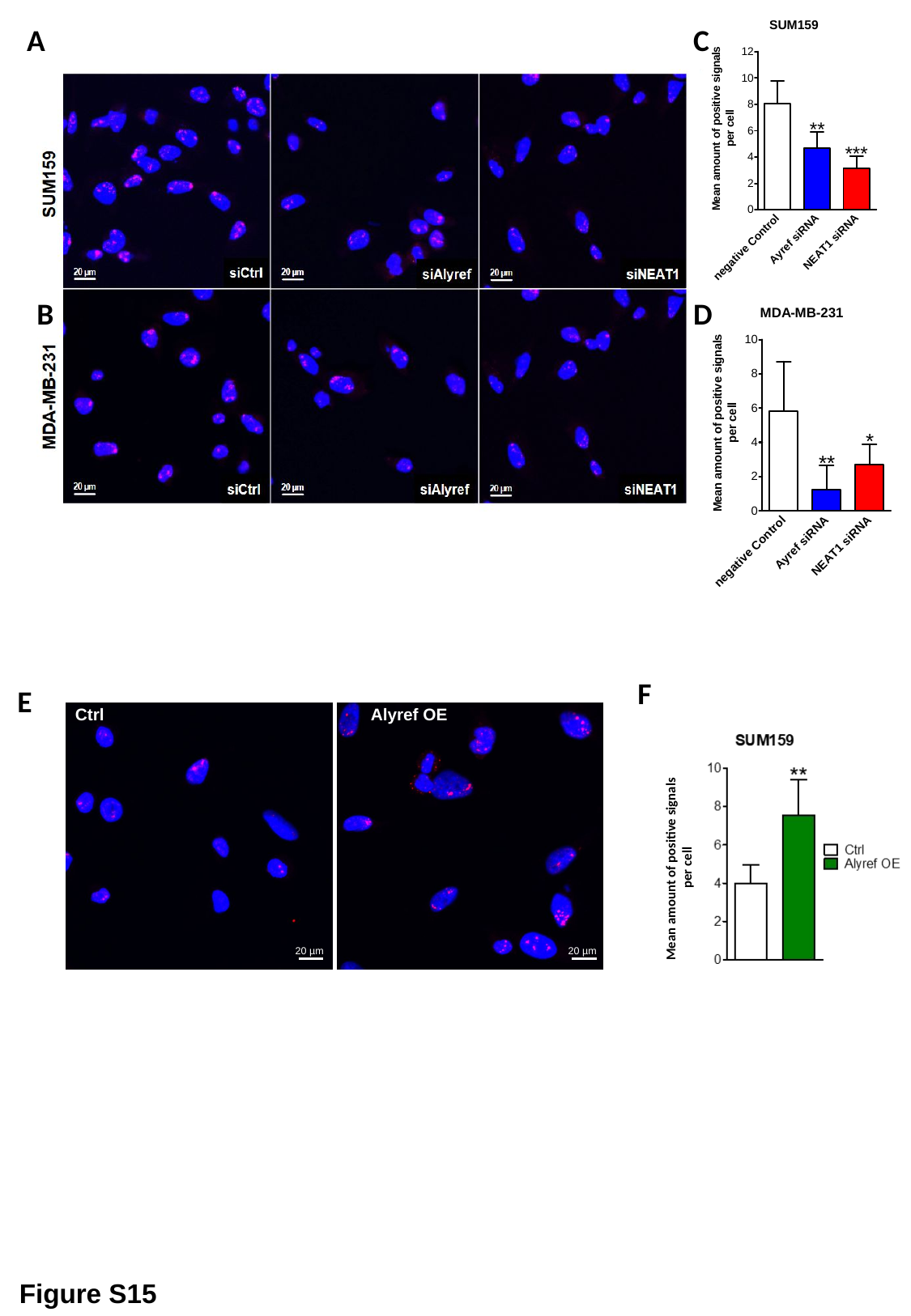

A
C
B
D
F
E
Mean amount of positive signals
per cell
Ctrl
Alyref OE
20 µm
20 µm
 Figure S15

## Slide 16
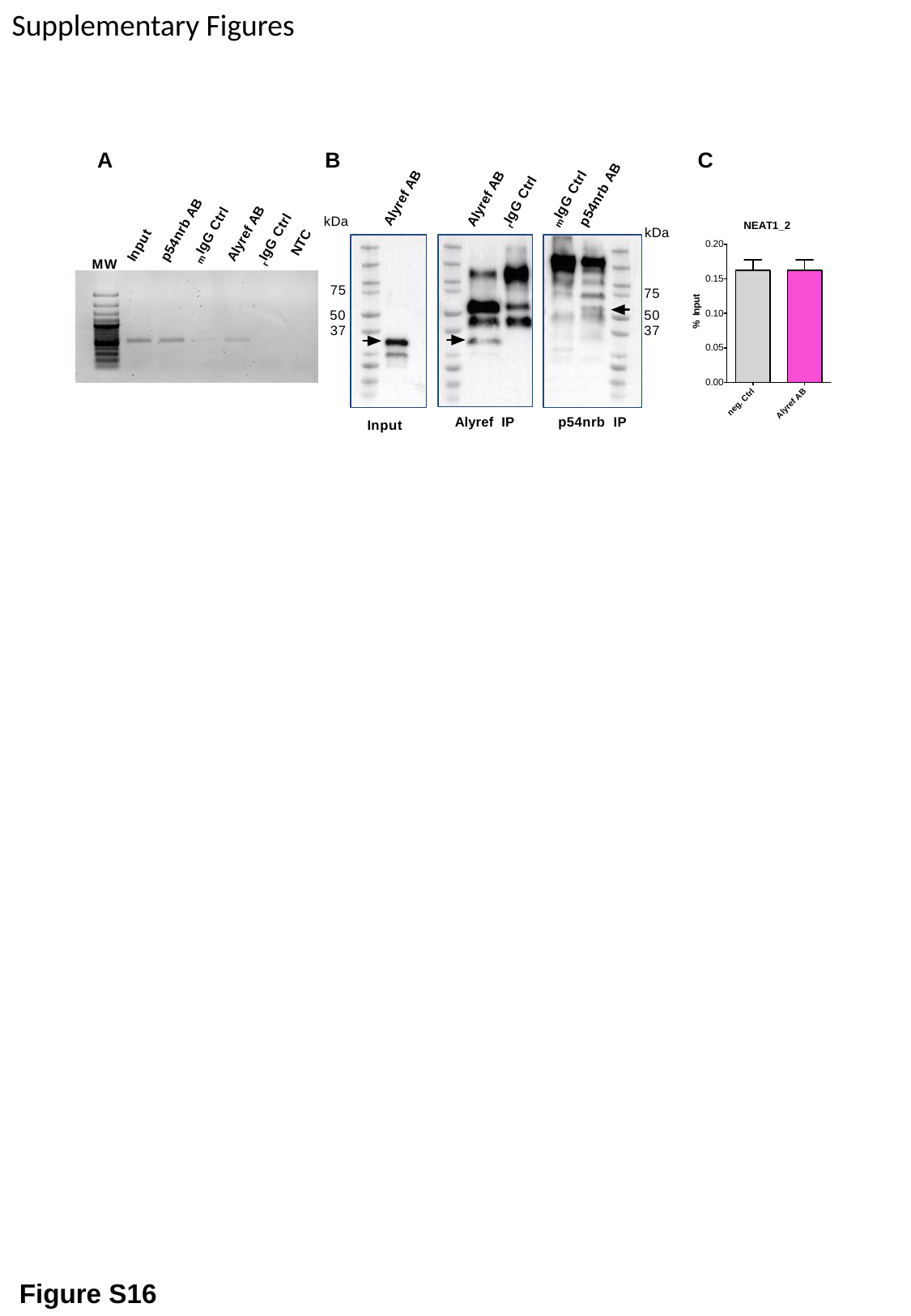

Supplementary Figures
 Figure S16

## Slide 17
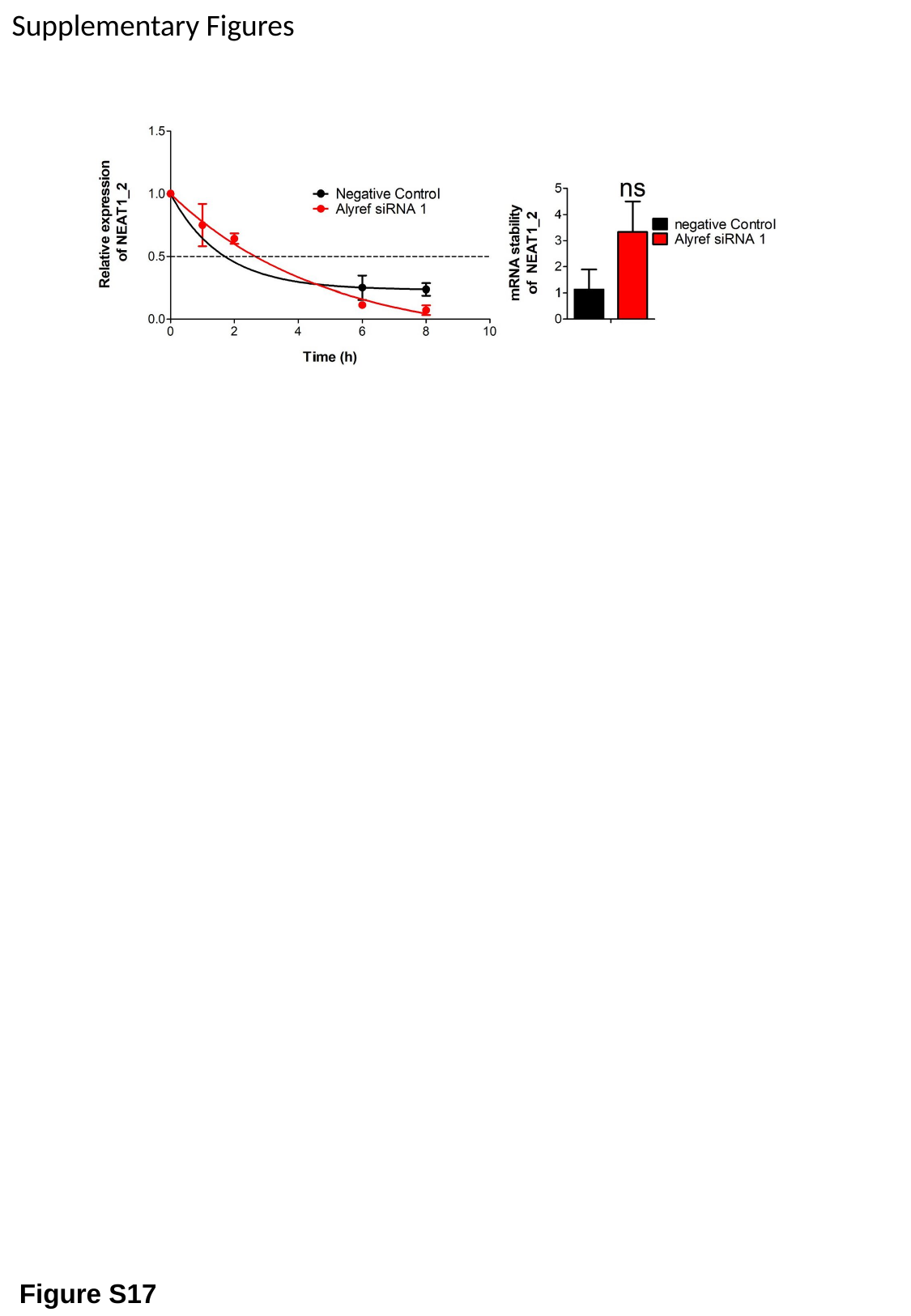

Supplementary Figures
 Figure S17

## Slide 18
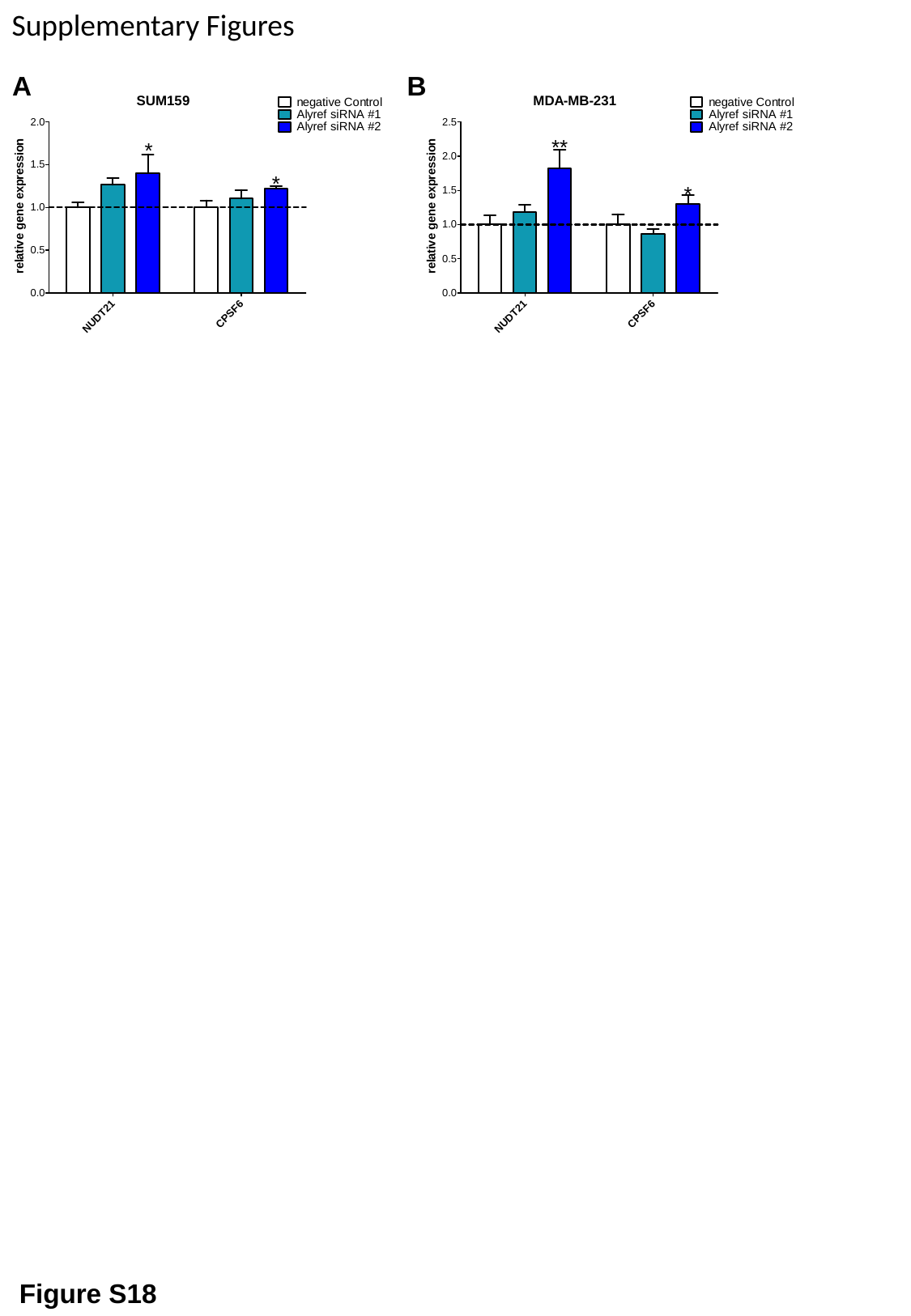

Supplementary Figures
A
B
 Figure S18

## Slide 19
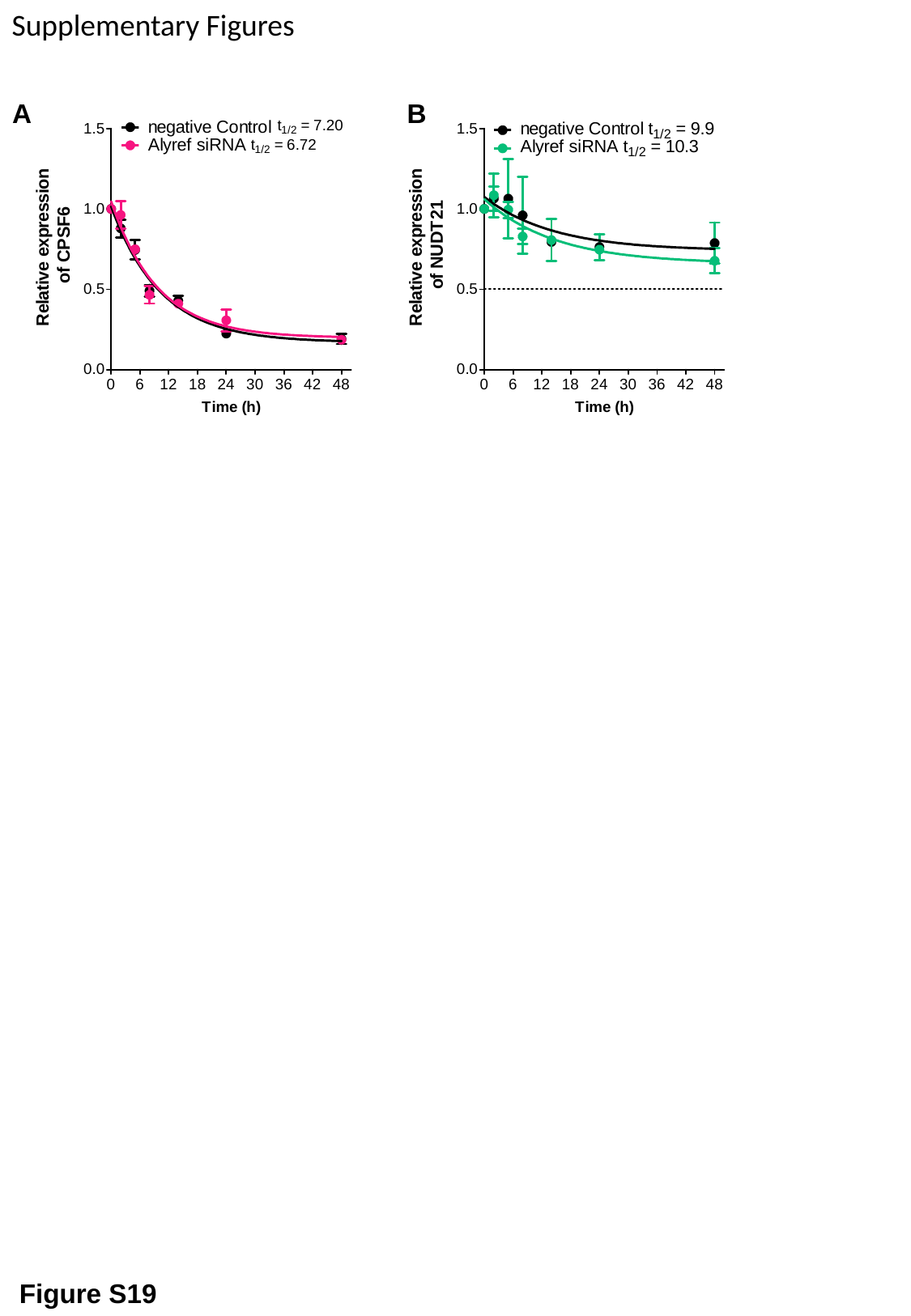

Supplementary Figures
A
B
 Figure S19

## Slide 20
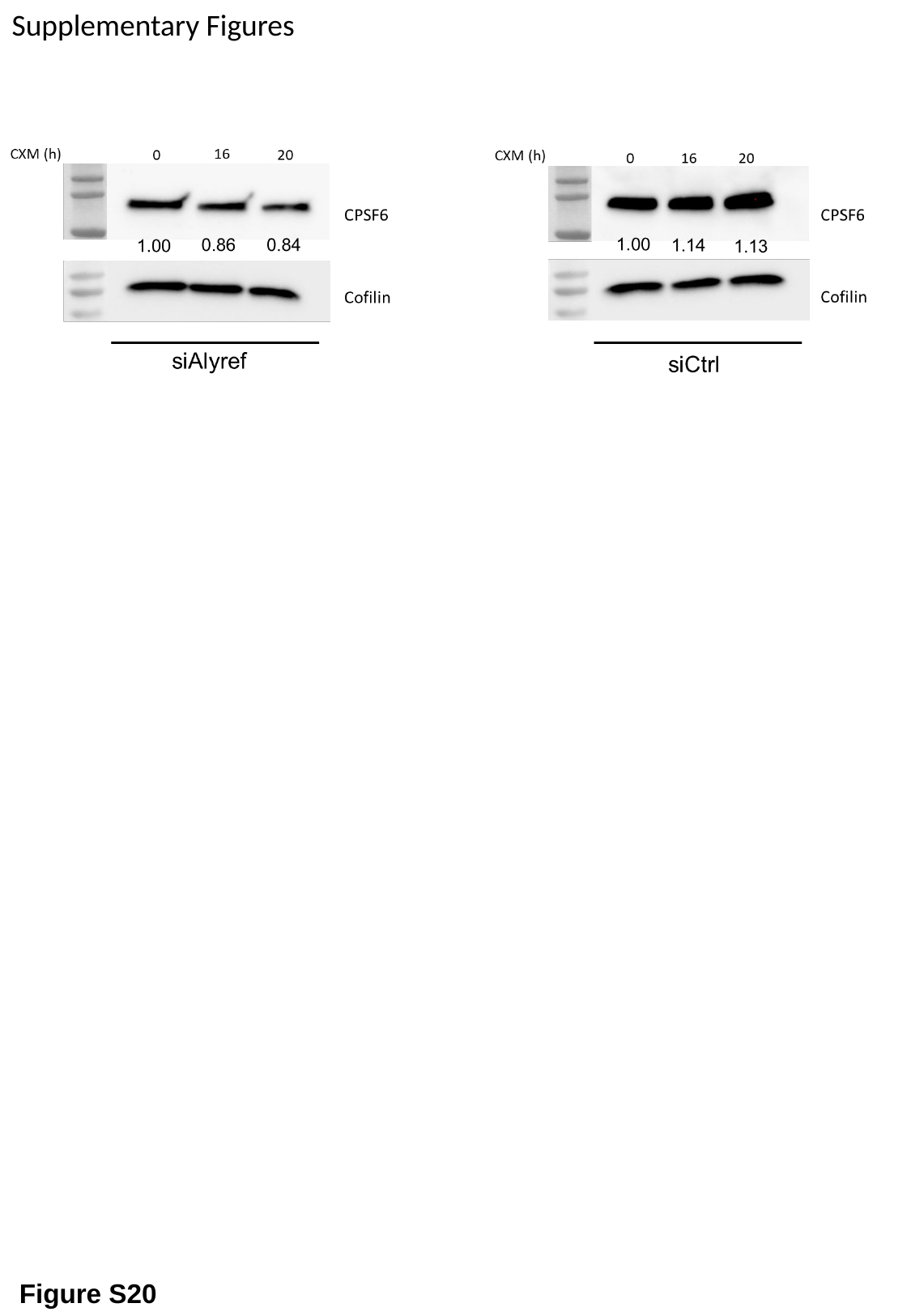

Supplementary Figures
 Figure S20

## Slide 21
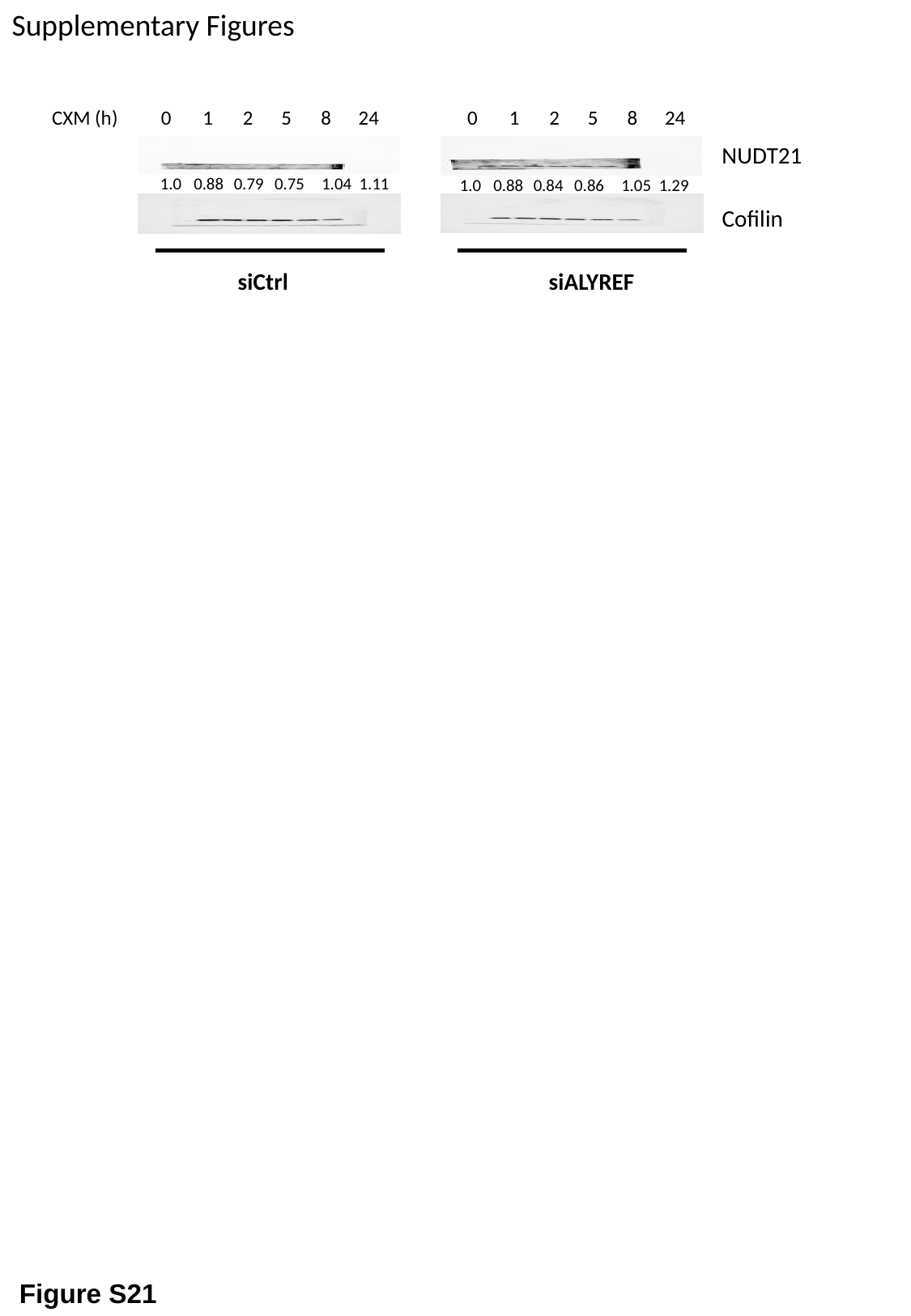

Supplementary Figures
CXM (h)
0
1
2
5
8
24
0
1
2
5
8
24
NUDT21
0.88
0.79
0.75
1.04
1.11
1.0
0.88
0.84
0.86
1.05
1.29
1.0
Cofilin
siALYREF
siCtrl
 Figure S21

## Slide 22
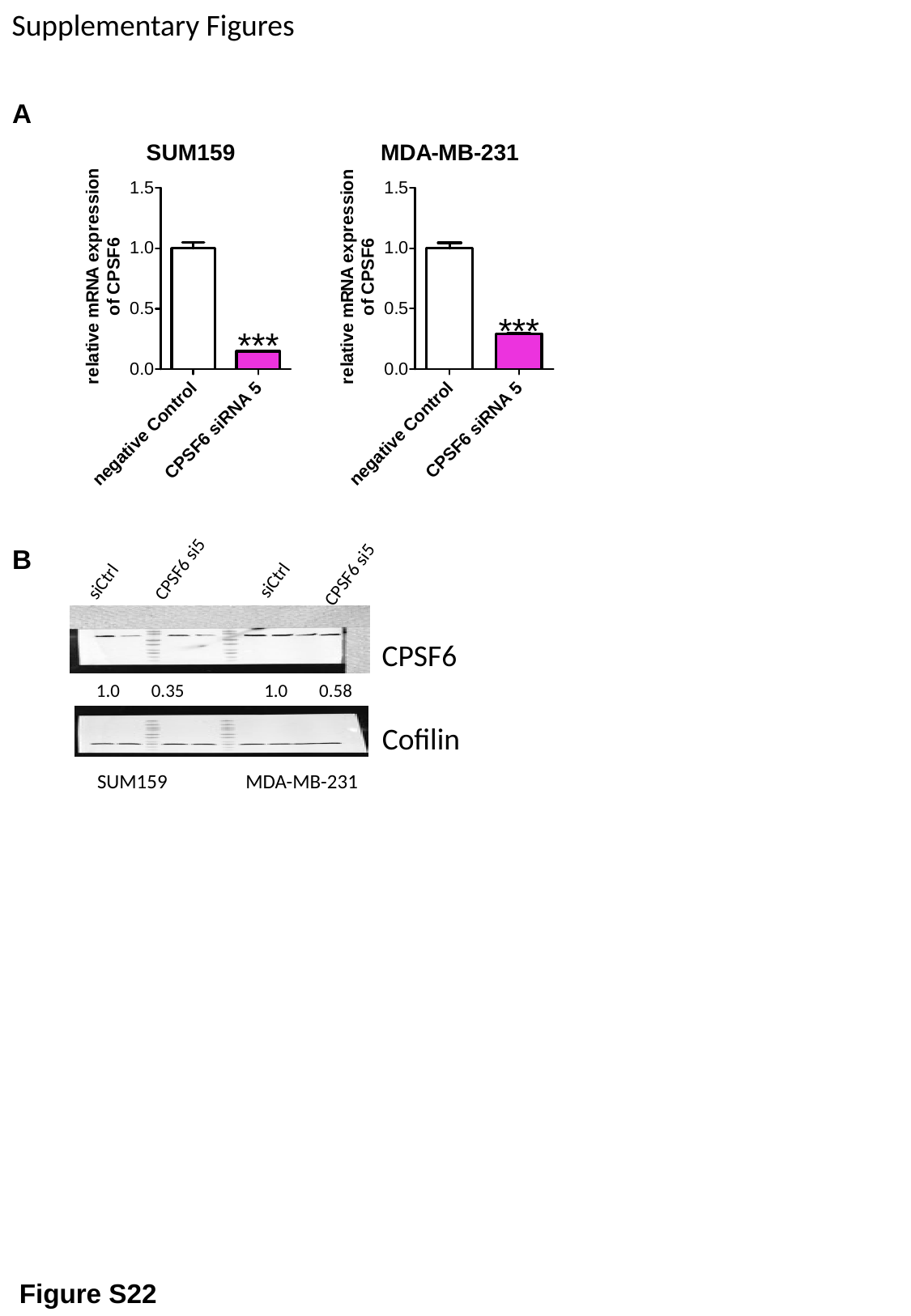

Supplementary Figures
A
B
CPSF6 si5
CPSF6 si5
siCtrl
siCtrl
CPSF6
1.0
0.35
1.0
0.58
Cofilin
SUM159
MDA-MB-231
 Figure S22

## Slide 23
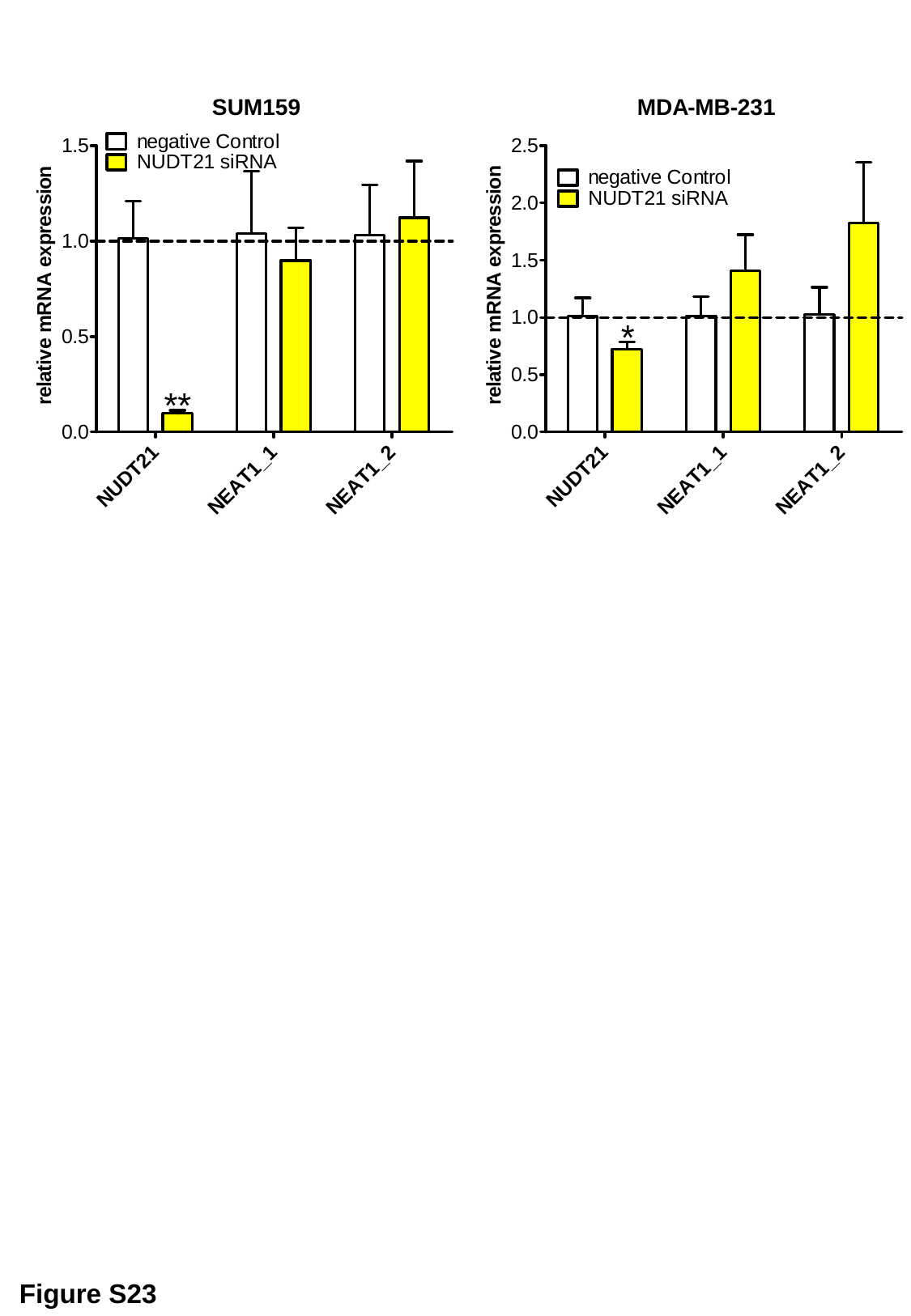

Figure S23

## Slide 24
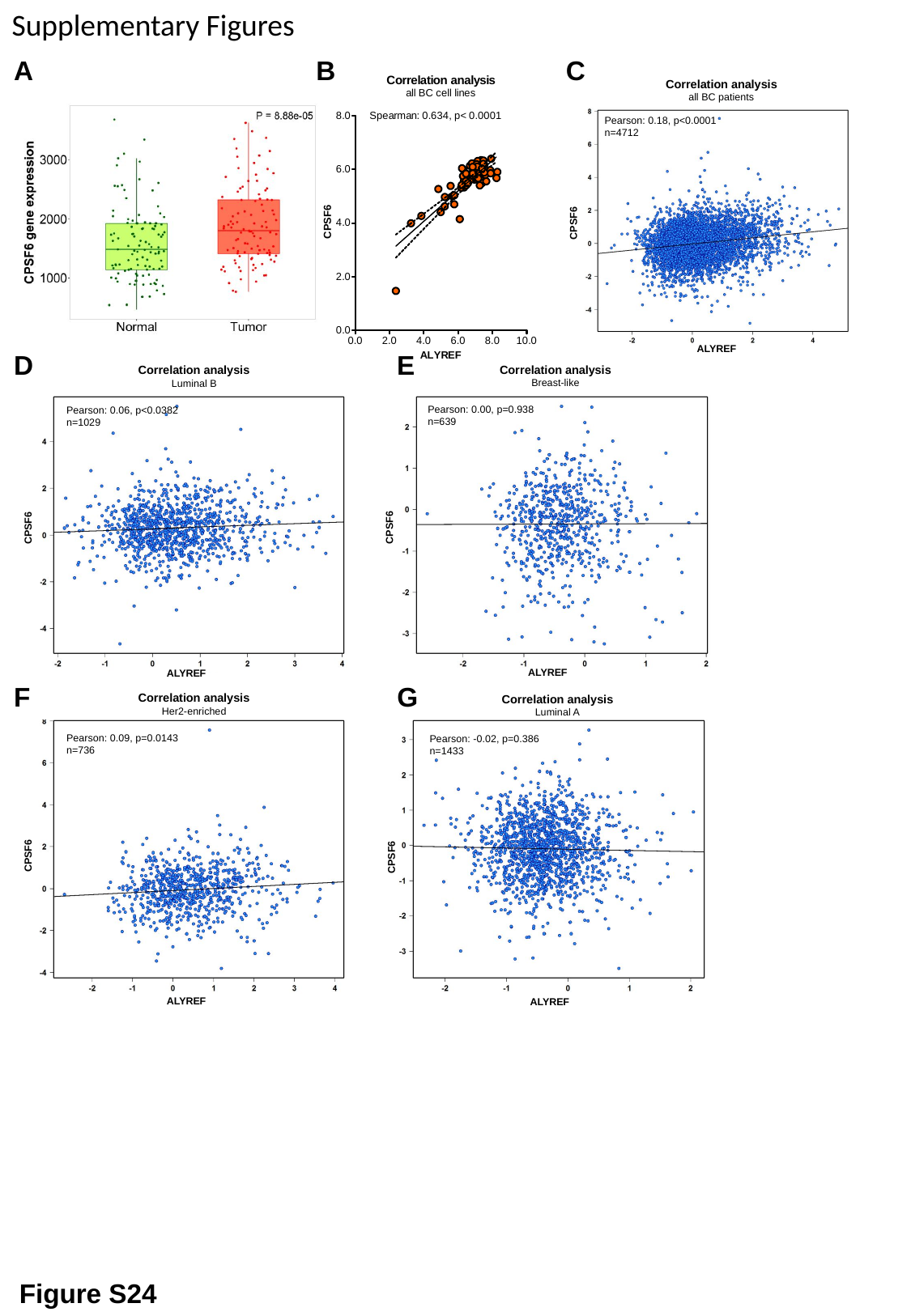

Supplementary Figures
A
B
C
Correlation analysis
all BC patients
Pearson: 0.18, p<0.0001
n=4712
CPSF6
ALYREF
D
E
Correlation analysis
Breast-like
Pearson: 0.00, p=0.938
n=639
CPSF6
ALYREF
Correlation analysis
Luminal B
Pearson: 0.06, p<0.0382
n=1029
CPSF6
ALYREF
F
G
Correlation analysis
Her2-enriched
Pearson: 0.09, p=0.0143
n=736
CPSF6
ALYREF
Correlation analysis
Luminal A
Pearson: -0.02, p=0.386
n=1433
CPSF6
ALYREF
 Figure S24
