# Supplementary material for: ALYREF, a novel factor involved in breast carcinogenesis, acts through transcriptional and post-transcriptional mechanisms selectively regulating the short NEAT1 isoform
Source: Cell Mol Life Sci. 2022 Jul 1;79(7):391. doi: 10.1007/s00018-022-04402-2 (PMC9249705; doi:10.1007/s00018-022-04402-2)
Supplement: Supplementary file 4 — Supplementary file4 (DOCX 41 KB) [file 18_2022_4402_MOESM4_ESM.docx]

**Table S1 Descriptive clinico-pathological parameters of the study cohort comprising of patients with breast cancer (n=128).**

| **Parameter** | **No. (%)** |
| --- | --- |
| **age at operation (yrs.)**  mean ± SD  median  minimum-maximum range | 60 ± 14.08  60.5  31.0-89 |
| **menopause**  pre-menopause  post-menopause | 34 (26.6)  94 (73.4) |
| **pathologic stage (TNM 2010)**  Stage I  Stage II  Stage III  Stage IV | 18 (14.1)  61 (47.7)  41 (32)  8 (6.3) |
| **Tumor grade**  G1  G2  G3  Missing information | 13(10.2)  54 (42.2)  48 (37.5)  13 (10.2) |
| **subtype**  invasive ductal carcinoma  invasive lobular carcinoma | 118 (92.2)  10 (7.8) |
| **type of surgery**  mastectomy  breast-conserving surgery  range | 95 (74.2)  33 (25.8)  0.10-9.20 |
| **Subtype (defined by immunohistochemistry)**  Estrogen receptor positive  HER2 positive  Trible negative (lack of ER, PR and HER2)  Missing values | 71 (55.5)  33 (25.8)  22 (17.2)  2 (1.6) |

**Legend**

ER: estrogen receptor

PR: progesterone receptor

**Table S2 Multivariate analysis of clinico-pathological parameters for the prediction of 5-year overall survival (OS) and 5-year disease free survival in patients with breast cancer in cohort 1 (n=128).**

| **Parameter** | **5-year overall survival** | | **5-year disease-free survival** | |
| --- | --- | --- | --- | --- |
|  | **HR (95%CI)** | ***p*-value** | **HR (95%CI)** | ***p*-value** |
| **Age at operation (yrs.)**  **Continuous variable** | 1.02 (0.98-1.06) | 0.381 | 1.01 (0.09-1.05) | 0.331 |
| **Tumor size**  **(continuous variable)** | 0.97 (0.72-1.30) | 0.861 | 1.06 (0.81-1.37)) | 0.658 |
| **stage (TNM 2010)**  **stage I**  **stage II**  **stage III**  **stage IV** | 1 (reference)  1.79 (0.21-15.3)  11.52 (1.1-120)  24 (1.83-315) | 0.592  0.041  0.015 | 1 (reference)  2.18 (0.27-17.6)  6.53 (0.75-56.81)  18.1(1.81-182.56) | 0.465  0.089  0.014 |
| **Subtype**  **Estrogen receptor positive**  **HER2 positive**  **Triple negative** | 1 (reference)  1.04 (0.33-3.23)  3.9 (0.89-16.9) | 0.936  0.069 | 1 (reference)  1.74 (0.68-4.4)  2.66 (0.82-8.59) | 0.243  0.101 |
| **Ki67 (continuous variable)** | 0.997 (0.97-1.01) | 0.762 | 0.998 (0.981-1.0116) | 0.838 |
| **ALYREF expression (mRNA levels)**  **<median**  **>median** | 1 (reference)  3.22 (1.25-8.24)) | 0.015 | 1 (reference)  2.72 (1.18-6.27) | 0.018 |

**Legend:**

HR: hazard ratio

95%CI: confidence interval

**Table S3 Statistical analysis of RNAseq data of ALYREF expression among breast cancer subtypes**

| **p(Welch) < 0.0001** | | | | |
| --- | --- | --- | --- | --- |
| RNAseq data, **ALYREF** expression according to Sorlie's subtypes | | | | |
| **Dunnett-Tukey-Kramer's test:** | | | | |
| gene-expression comparisons | | | p-value | |
| HER2-E | **<** | Basal-like |  | < 0.0001 |
| Luminal A | **<** | Basal-like |  | < 0.0001 |
| Luminal A | **<** | HER2-E |  | < 0.0001 |
| Luminal B | **<** | Basal-like |  | < 0.0001 |
| Luminal B | **>** | HER2-E |  | < 0.0001 |
| Luminal B | **>** | Luminal A |  | < 0.0001 |
| Normal breast-like | **<** | Basal-like |  | < 0.0001 |
| Normal breast-like | **<** | HER2-E |  | < 0.0001 |
| Normal breast-like | **<** | Luminal A |  | < 0.0001 |
| Normal breast-like | **<** | Luminal B |  | < 0.0001 |
|  | | | | |

**Table S4 Statistical analysis of RNAseq data of CPSF6 expression among breast cancer subtypes**

| **p(Welch) < 0.0001** | | | | |
| --- | --- | --- | --- | --- |
| RNAseq data, **CPSF6** expression according to Sorlie's subtypes | | | | |
| **Dunnett-Tukey-Kramer's test:** | | | | |
| gene-expression comparisons | | | p-value | |
| HER2-E | **<** | Basal-like |  | < 0.0001 |
| Luminal A | **>** | HER2-E |  | < 0.0001 |
| Luminal B | **>** | HER2-E |  | < 0.0001 |
| Normal breast-like | **<** | Basal-like |  | < 0.0001 |
| Normal breast-like | **<** | Luminal A |  | < 0.0001 |
| Normal breast-like | **<** | Luminal B |  | < 0.0001 |
| Luminal A | **<** | Basal-like |  | < 0.01 |
| Luminal B | **>** | Luminal A |  | < 0.10 |
| Luminal B | **=** | Basal-like |  | > 0.10 |
| Normal breast-like | **=** | HER2-E |  | > 0.10 |
|  | | | | |
